# Feasibility of a Dielectric Elastomer Augmented Aorta

**DOI:** 10.1002/advs.202001974

**Published:** 2021-01-25

**Authors:** Morgan Almanza, Francesco Clavica, Jonathan Chavanne, David Moser, Dominik Obrist, Thierry Carrel, Yoan Civet, Yves Perriard

**Affiliations:** ^1^ Integrated Actuators Laboratory École Polytechnique fédérale de Lausanne (EPFL) Neuchâtel 2000 Switzerland; ^2^ ARTORG Center for Biomedical Engineering Research University of Bern Bern 3012 Switzerland; ^3^ Department of Cardiovascular Surgery University Hospital and University of Bern Bern 3012 Switzerland

**Keywords:** artificial muscles, augmented aortas, cardiac assist devices, dielectric elastomer actuators

## Abstract

Although heart transplantation is a gold standard for severe heart failure, there is a need for alternative effective therapies. A dielectric‐elastomer aorta is used to augment the physiological role of the aorta in the human circulatory system. To this end, the authors developed a tubular dielectric elastomer actuator (DEA) able to assist the heart by easing the deformation of the aorta in the systole and by increasing its recoil force in the diastole. In vitro experiments using a pulsatile flow‐loop, replicating human physiological flow and pressure conditions, show a reduction of 5.5% (47 mJ per cycle) of the heart energy with this device. Here, the controlled stiffness of the DEA graft, which is usually difficult to exploit for actuators, is perfectly matching the assistance principle. At the same time, the physiological aortic pressure is exploited to offer a prestretch to the DEA which otherwise would require an additional bulky pre‐stretching system to reach high performances.

## Graphical abstract

A dielectric‐elastomer aorta is used to augment the physiological role of the aorta in the human circulatory system. In vitro experiments show a reduction of 5.5% of the heart energy. The controlled stiffness of the dielectric‐elastomer, which is usually difficult to exploit for actuators, is perfectly matching the assistance principle.

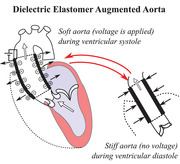

## 1 Introduction

Heart failure (HF) is a devastating disease that affects more than 11 million people in the United States and Europe and more than 23 million worldwide.^[^ ^1^ ^]^ The first successful attempt to treat HF with an artificial assist device in humans dates back to 1966.^[^ ^2^ ^]^ Although heart transplant is the gold standard for selected patients suffering from severe heart failure, there is a need for alternative effective therapies due to the shortage of heart donors. Further developments of cardiac assist devices could eliminate or delay the need for a transplant.

Technological solutions to assist the heart (Text S1, Supporting Information) can be divided into two families of devices: ventricular assist devices (VADs) and aortic counterpulsation devices. On the one hand, current VADs are based on rotary pumps (axial and centrifugal pumps) characterized by a single rotating element which creates a constant flow.^[^ ^3^ ^]^ On the other hand, the basic principle of aortic counterpulsation allows an increase of coronary blood flow (during diastole) and unloads the left ventricle (during systole).^[^ ^4^ ^]^ In the intra‐aortic balloon pump (IABP, the most common aortic counterpulsation device), a balloon is inflated inside the aorta during the diastolic phase of the heart to increase aortic pressure (which leads to increased coronary flow) while presystolic deflation of the balloon decreases the afterload pressure. Aortic counterpulsation device avoids rotary components (associated with high risk of haemolysis and thrombosis which force patients to use anti‐coagulants during their lifetime^[^ ^5^ ^]^) and it ensures pulsatile flow (which favours capillary perfusion^[^ ^6^ ^]^). However, IABP equipment, being pneumatically driven, are bulky and difficult to fully implant.

Two decades ago, Pelrine^[^ ^7^ ^]^ introduced a new family of soft actuators called dielectric elastomer actuator (DEA). Those DEAs are made of a hyper‐elastic membrane sandwiched between compliant electrodes. When subjected to an electric field, the generated Maxwell stress causes the membrane to compress out‐of‐plane and to expand in‐plane. Typical features of dielectric elastomer actuators are: large deformation beyond 100%, fast response, and low weight.

After two decades of research on DEAs, mainly oriented toward linear actuator or haptic devices, it is still hard to reap their potential. First, an initial pre‐stretching of the membrane^[^ ^7^ ^]^ is required to withstand the high electric field necessary for a large deformation. Second, DEAs intrinsically behave more like a controlled stiffness spring than a conventional actuator. Their native driven compliance behavior results in a complex force‐displacement working area, resembling as a “banana shape (Figure S23, Supporting Information)” instead of a square domain commonly used for classic actuators.^[^ ^8^ ^]^ To apply the pre‐stretching or to morph the area of operation to more conventional ones, external mechanical systems are needed, such as positive or negative stiffness springs.^[^ ^9^, ^10^ ^]^ Those passive elements add mass and complexity to the design, limiting the advantages of DEA.

## 2 Methods

In the present study, we present the first dielectric‐elastomer‐augmented aorta (DEAA). This DEAA consists of a tubular DEA with an electrically driven compliance. It is designed to assist the pulsatile nature of the heart by means of an innovative aortic counter‐pulsation approach. The possibility of an electrically driven counterpulsation device paves the way for a fully implanted device (not possible for current pneumatically driven aortic counterpulsation devices) and for a high‐level cardiac assistance.

DEA pumps have already been developed in the past (Text S2, Supporting Information). However, the actuator and its application has to be thought as a whole to reveal its full potential. Since a tubular DEA replaces the ascending aorta, it exploits directly the controlled stiffness of DEAs and by doing so it reaps the full benefit of DEAs. To the best of our knowledge, no attempt of implanting these actuators in human or animals as hemodynamic support / partial replacement of the muscle function has been reported so far. Therefore, we aim at exploiting this technology for medical applications starting from an assist device for the failing heart.

**Figure 1:**
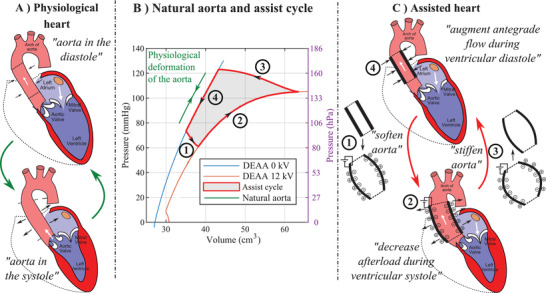
Working principle of a dielectric‐elastomer‐augmented aorta (DEAA) for cardiac assist device. A) Physiological behavior of the heart highlighting the diastolic and systolic phases. B) Pressure–volume characteristic of a biological aorta and of an DEAA. The green line shows a physiological cycle also corresponding to a cycle operated without assistance. The black line shows the assisted cycle with the corresponding steps. The area delimited by 1‐2‐3‐4 corresponds to the energy provided by the DEAA. C) DEAA working principle: 1) at end of the diastole, the voltage is applied and the DEA expands; 2) during the systole, the pressure increases leading to an expansion of the DEA; 3) at end of the systole, the voltage is removed and the DEA contracts; 4) during the diastole, the pressure decreases leading to a contraction of the DEA.

A physiological aorta in healthy patients stores elastic energy in the systole and releases it during the diastole as indicated by the green curve in **Figure** **1**. It is known as the Windkessel effect.^[^ ^11^ ^]^ Despite the pulsatile nature of the cardiac pumping, the compliance of the aorta ensures a rather constant peripheral blood flow (Windkessel effect). In our assist device, the tubular DEA physiologically replaces a part of the ascending aorta, as shown in Figure 1C, to unload the left ventricle which provides most of the heart energy. In the assisted operation, the stiffness/compliance is electrically controlled to have different values at expansion or contraction phase (steps 2 and 4 in the cycle, Figure 1). By providing energy, this tubular dielectric elastomer (actuator) augments the role of the aorta with its unique driven compliance.

At the beginning of the systolic phase, voltage is applied (steps 1, Figure 1) to soften the DEAA, allowing it to expand as the ventricle ejects blood out of the aortic valve (steps 2, Figure 1). This expansion also decreases the afterload seen by the ventricle. At the beginning of diastole, the aortic valve closes, the voltage is switched‐off (steps 3, Figure 1) causing the DEAA to return to its contracted shape, thereby augmenting forward flow (steps 4, Figure 1). The DEAA contracts first because of the vanishing of the voltage and then because of the decrease of the aortic pressure (diastole). The area of the cycle (delimited by 1‐2‐3‐4, Figure 1) is the energy provided by the augmented aorta to the fluid (blood).

In this working principle, the DEA benefits, in terms of performance, from an adequate operating condition. First of all, the pressure variation, induced by the heart in the aorta, allows the DEA to circumscribe the cycle (through a difference of compliance when the pressure increases or decreases). Second, the aforementioned pre‐stretching for DEA is provided by the aortic pressure (no additional complex elements, lowering the energy density, are required).

Assuming that the energy provided by the heart is around 0.8 J, producing a DEAA able to provide ≈5–10% of the heart energy (40–80 mJ) is a milestone. Multi‐layer tubular silicone DEAs are required to reach enough energy for assisting the heart and reduce the working voltage. Most tubular DEAs that are fabricated and studied^[^ ^12^, ^13^ ^]^ are based on rolled acrylic (VHB from 3M) films to obtain a tubular structure. For the fabrication of multi‐layer tubular DEAs based on silicone, only two processes exist: deep coating^[^ ^14^ ^]^ and rolling film^[^ ^15^ ^]^.

In the development of a soft cardiac‐assist‐device, we replace and augment the role of the ascending aorta with a tubular DEA. To this end, we developed a process to achieve a reliable and highly efficient multilayer tubular DEA from commercial silicone films. Since the tubular DEA is designed to be implanted, we confined the electric field (100 V.μm^−1^) inside the DEA tube through an electrical shield which avoids any electrical field in the surrounding tissue. To fully characterize the assistance of the DEAA in terms of energy provided, we measured the pressure–volume characteristic using a custom‐made test bench. Finally, to study the complex interaction between the DEAA and the cardiovascular system, we tested our device in a pulsatile flow‐loop replicating human physiological flow and pressure conditions.

### 2.1 Fabrication of the Tube

**Figure 2:**
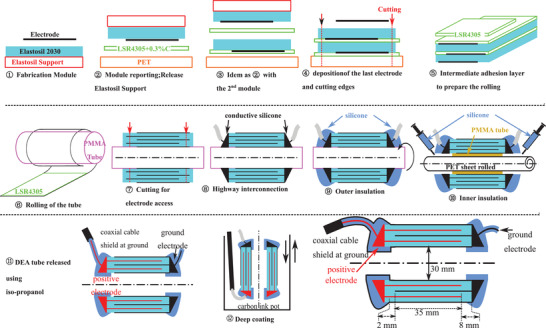
Manufacturing process from a commercial film to a tubular dielectric elastomer actuator. 1) Deposition of the carbon base electrode on top of a silicone film to obtain a module. 2) The module is placed up‐side down on a PET support with a silicone adhesion layer (LSR4305+0.3%C). 3) A second module is placed on top of the previous stack. 4) A final electrode is deposited. 5) A deposition of a silicone layer is done on the lower half of the stack to glue the layers of the tube in (6) when the tube is rolled around a PMMA tube. 7) The tube is cut across the whole thickness on both extremities to reach the different electrodes. 8) A conductive silicone (LR 3162) is deposited to connect the electrodes embedding a high voltage cable. 9) Outer insulation with silicone (Sylgard 186) is done. 10) The inner insulation (Sylgard 186) is done using a protective PMMA tube and PET sheet. 11) The tube is released from its support. 12) Carbon ink is finally deposited by deep coating to ensure electric shielding and the final tube is obtained

The need of films with constant properties over wide surfaces encourages the selection of industrially produced film. A brand new process is proposed, regarding the aforementioned film, which are very soft and sold attached to a dedicated support. The fabrication process, described in **Figure** **2**, is based on stacking and rolling commercial films followed by electrical interconnections, safety encapsulation and shielding.

**Figure 3:**
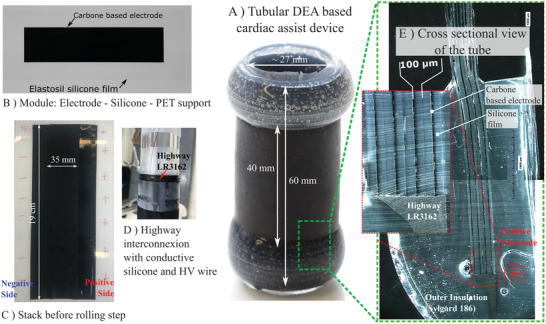
Fabrication results of the DEAA ‐ tubular DEA with eight layers 100 µm thick. A) Photo of the final device. B) Photo of the initial module (electrode–silicone–PET support). C) Photo of the multilayer structure right before the rolling step. Negatively and positively charged sides are annotated. D) Highway interconnection realization ongoing. The high voltage wire is embedded in the conductive silicone (LR 3162). E) Cross sectional view of the different layers under optical microscope. We can distinguish the eight layers of 100 µm thick Elastosil film, the carbon based electrode, the highway interconnection (LR 3162), and the outer insulation (Sylgard 186).

First a carbon‐based electrode is deposited on top of a silicone film (Elastosil film 2030 200 µm) with its support to make a module (① in Figure 2 and in **Figure** **3**B. In panels ② and ③, thanks to a silicone based adhesion layer,^[^ ^16^ ^]^ the silicone film and its electrode are successively transferred up‐side down from their initial support to the stack (the first layer being on a polyethylene terephthalate [PET] substrate). The silicone‐based adhesion layer^[^ ^16^ ^]^ made of carbon black powder and silicone also smooths the field concentration and improves the withstand voltage. After the deposition of the last electrode, the stack (Figure 3C) is then cut (④ in Figure 2). The active area is 190 mm long and 35 mm large. The stack is rolled (⑥ in Figure 2) around a tube of 30 mm of diameter, corresponding to the size of human aorta at rest, after a deposition of silicone to glue the layers stacked during the rolling (⑤ in Figure 2).

In ⑦, the tube is cut at the edges to access electrodes. In ⑧, the interdigited electrodes are interconnected with conductive silicone and a wire is introduced (Figure 3D ). It behaves as a highway to distribute the charges all along tube's edges. In fix circled numbers ⑨ and ⑩, silicone is added around the electrical interconnection to make inner and outer insulations.

Finally, an electric shield is deposited by deep coating (⑪ in Figure 2). This electric shield, connected to the ground, is a conductive layer enclosing the DEA. Since positive electrodes as well as their contacts are completely embedded in the shield, the electric field is confined in the dielectric structure. Therefore, once implanted, there is very low risk to have a field or current leakage in physiologic tissues (conductive and with high dielectric constant).

With the tube's inner diameter of 30 mm (in the active area) and a total length around 60 mm (Figure 3), which can be reduced to 50 mm with a lower safety distance for insulation, the DEA could replace part of the ascending aorta of human, which is 8 cm long and circa 30 mm wide in diameter at rest.^[^ ^17^ ^]^

**Figure 4:**
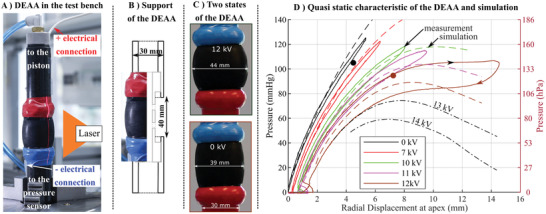
Set‐up and quasi‐static measurements of the dielectric‐elastomer‐augmented aorta (DEAA)–tubular DEA with four layers of 200 µm. A) DEAA under tests: The pressure, the volume, the voltage, and the current of the tube are measured. B) Sectional view of the mechanical set‐up to maintain the tube between two coaxial tubes. Through the radial holes, the tube is inflated with air. C) Deformation of the tube pressurized around 100 mmHg when the voltage is on (12 kV) and off (0 kV), (measurements are in Figure S15, Supporting Information). D) Pressure–displacement characteristic of the DEAA at different voltages according to measurements (full line) and computational finite‐elements simulation (dash line). The measurements are done in quasi‐static conditions. Radial displacement is measured by a laser line profile sensor. Brown and black points correspond to the two states of the DEEA of Figure 4C.

Aorta presents a complex mechanical behavior which depends on the age, the patient, and potential pathology.^[^ ^18^, ^19^, ^20^, ^21^, ^22^ ^]^ Advance composite material,^[^ ^23^, ^24^, ^25^, ^26^ ^]^ could reproduce the strain‐stiffening behavior of a natural aorta, that is, the typical J‐shaped at 20–30% of deformation (Figure S23, Supporting Information). This J‐shaped ensures the mechanical stability (aneurysms and “blowout”) in any highly distensible pressure vessel.^[^ ^27^, ^28^, ^29^ ^]^ Without this J‐shaped, analysis of the mechanical stability in the pressure–volume characteristic, as proposed in **Figure** **4**, is mandatory. In this work, the number of layers, that is, the total thickness, has been chosen to mimic the average mechanical behavior of a real ascending aorta in a physiological range of pressures (Figure S24, Supporting Information).

The electrical impedance, measured in the initial state at a low voltage, is equivalent to a resistance of 10*k*Ω in series with a capacitor of 1.6 nF giving a time response of dozens of microseconds, which is fast enough compared to the dynamics of the heart.

### 2.2 Characterization of the Dielectric‐Elastomer‐Augmented Aorta

A custom‐made test bench has been built (Figure 4A and Movie S1, Supporting Information), to measure the pressure–displacement characteristic of the DEAA (tubular DEA) (Figure 4D). For this purpose, the DEAA has been placed in between two coaxial tubes (Figure 4B) and a pneumatic set‐up imposes the pressure of the tube while an amplifier controls the voltage. A laser line profile sensor measures the radial displacement of the tube. The pressure–displacement characteristic shows up to 14 mm of displacement at 12 kV. Quasi‐static measurement over a full span of pressure and high voltages enlarge the hysteresis. Indeed, at a fixed voltage, the Maxwell pressure increases when the film is squeezed what causes an amplification of the hysteresis.

At 12 kV (brown line in Figure 4D), after reaching 105 mmHg, there was a plateau: the volume of the tube increases while the pressure remains constant. Our simulation even indicated the pressure would even decrease for an increase of volume. This behavior is not observable with a test bench imposing pressure as it leads to a plateau (snap‐through). Few characterizations where the constant product (pressure × volume , Boyle's law) is small confirm this decrease in the curve pressure‐volume (Figure S14, Supporting Information).

Static 2D axisymmetric finite element simulations shows a good agreement with measurements (Figure 4D). A perfect estimation of the behavior is difficult, this is because the elastomer exhibits a hyperelastic behavior and a strong dependence to its history^[^ ^30^ ^]^ (Mullins effect Figure S22, Supporting Information). Simulation in Figure 4D is used to illustrate the behavior of the DEAA in the unstable region where the volume/displacement increases while the pressure decreases (slope inversion). A reduced strain‐stiffening (J‐shaped) elastomer response accentuated by an applied electrical field has promoted this instability.

In general, working with the highest voltage increases the displacement and so the energy that will be transferred to the fluid (Figure 4D). However, working at too high voltage increases the risk of breakdown. In addition, imposing the pressure while working above a certain voltage leads to instabilities,^[^ ^31^ ^]^ thus increasing the risk of premature failure. According to our experiments, working in a reduced pressure range (up to 105 mmHg) at 12 kV appears to be a safe operating voltage, while still reaching high displacement, and thus high energy, thanks to the vicinity of the plateau (Figure 4D).

## 3 Results

### 3.1 Interaction between the Dielectric‐Elastomer‐Augmented Aorta and a Fluid

**Figure 5:**
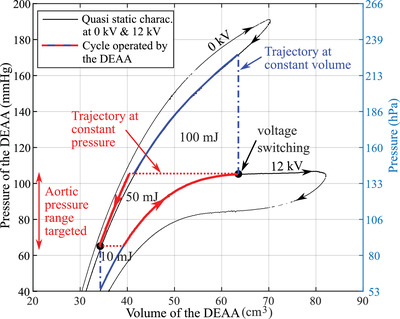
Quasi static pressure–volume characteristic of the DEAA at 0 at 12 kV. The bold lines highlight the trajectories at constant voltage: in red when there is a pressure balance between the DEA and the rest of the aorta and in blue when there is a pressure unbalance. The red dash lines show the transition considering a slow electrical switching, that is, pressure balance. The blue dash dot lines give the path followed in case of a fast voltage switching. The working human aortic pressure change targeted with the proposed actuator is indicated since it is slightly different from an healthy patient. The energy associated to the different area are also indicated.

The mechanical work provided by the DEAA is the work of the pressure forces all along the interface between the fluid and the membrane of the DEA. For a homogeneous pressure in the lumen of DEAA, the work becomes related to the area of the pressure–volume cycle in the **Figure** **5**. From the displacement measured along the tube with the laser line profile sensor and considering axisymmetric deformation, the volume of the DEAA is estimated. In working conditions, the interaction between the DEA and the pulsating human flow will define operating points (pressure–volume) that will describe the cycle. Quasistatic measurements gives a preliminary estimation of the energy that a DEAA will provide when interacting with human cardiovascular system.

At constant voltage (0 or 12 kV), the operating point moves along the quasi static characteristic of the DEAA according to the voltage applied. A slow voltage switching, regarding the dynamic of the flow, keeps a pressure balance along the aorta. Meanwhile the change of aorta pressure being still slower than this switching, the pressure remains constant during the switching (constant pressure trajectory, red dot line in Figure 4). As soon as the dynamic of the voltage switching is faster than the dynamic of the flow, the flow is not fast enough to compensate the drastic change of local pressure in the lumen of the DEAA; therefore, the volume does not change. After the transient time, the DEAA follows the pressure imposed by the cardiovascular system (full red lines in Figure 5) since the volume of the DEAA is four times smaller than the one of the heart. In that case, the DEAA follows a constant volume (CV) trajectory (blue dash‐dot line in Figure 5). These constant pressure (CP) and constant volume (CV) trajectories circumscribe possible trajectories. They respectively shape the CV–CP (constant voltage–constant pressure) and the CV–CV (constant voltage–constant volume) cycles.

The hysteresis, which depends on the frequency, will affect the cycle even though not working on the full range of pressure would reduce its influence. Test bench experiments on the assist CV–CP cycle confirms a cycle close to the two closest branches of the hysteresis loop as drawn by the red curve in Figure 5 (Figure S13, Supporting Information). The CV–CP cycle provides mechanical work of 50 mJ per cycle, roughly 6% of the energy provided by the heart and the CV–CV cycle goes up to 160 mJ, that is, 18% of the energy provided by the heart.

### 3.2 Dielectric‐Elastomer‐Augmented Aorta in Pulsatile Flow‐Loop Replicating Physiological Aortic Pressure and Flow

**Figure 6:**
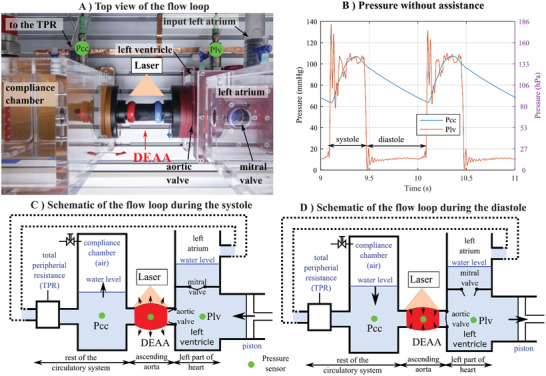
Pulsatile flow‐loop replicating physiological flow and pressure conditions. A) Top view of the flow loop focusing on the DEAA in between the left ventricle and the compliance chamber. B) Temporal signal of the pressures in the compliance chamber (Pcc) and the left ventricle (Plv). Systolic and diastolic phases are annotated. Schematic side view of flow loop's behavior (piston, valves) and DEAA deformation during systole (C) and diastole (D) with the state of the valves.

Once implanted in the ascending aorta, the DEAA will interact with a complex fluid dynamic system including valves, the left ventricle, and the rest of the aorta. To test our device close to real working conditions and estimate the energy that effectively assists the heart, we built a pulsatile flow‐loop replicating physiological flow and pressure conditions^[^ ^32^, ^33^ ^]^ (Movie S3, Supporting Information). This mock‐up, so called “flow loop” (**Figure** **6**A) is designed to mimic the pressure and flow inside the ascending aorta (i.e., where tubular DEA is placed). There are three main parts (Figure 6C): i) the left part of the heart (left atrium and ventricle), ii) the ascending aorta, and iii) the remaining circulatory system. A piston, moving back and forth cyclically (Figure S20, Supporting Information), reproduces the contraction of the left ventricle. The valves are mechanical valves clinically used for valve replacement in patients. The DEAA replaces the ascending aorta, just after the aortic valve. The remaining circulatory system is made of a compliance chamber used to mimic the compliance of the arteries and a flow resistance used to mimic the total peripheral resistances (i.e., the resistance of the microvascular network). Pressures are measured in the compliance chamber (Pcc), in the lumen of DEA (PDEA), and in the left ventricle (Plv). The operating points between 65 and 105 mmHg (Figure 6B) were tuned to be in the range of physiological pressure while avoiding the aforementioned unstable area of DEA.

**Figure 7:**
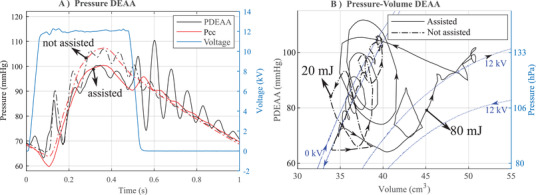
Effect of the DEAA in a pulsatile flow‐loop replicating physiological flow. A) Pressure in the DEAA and in the compliance chamber with (assisted, continous lines) or without (not assisted, dash‐dotted lines) voltage. B) Pressure‐volume of the DEAA in the flow loop with the energy provided and the quasi static measurement of the DEAA at 0 and 12 kV (blue dash line).

DEAA voltage follows the profile of the **Figure** **7** with a rising time and falling time of 50 ms (around ten times faster than the dynamic of the left ventricle). The voltage was activated when the left ventricle (piston) starts to contract and deactivated 10 ms after it starts to expand (Figure S20, Supporting Information). The application of the voltage increases the aortic deformation as expected (Movie S4, Supporting Information). Electrical based estimation (350 mJ charge ‐ 126 mJ discharge = 224 mJ transferred) is not accurate because the DEA has losses both from the mechanical part and from the electrical part.^[^ ^16^ ^]^ A direct approach consists of estimating the mechanical work done by the DEAA. For this purpose, a pressure probe (0.36 mm diameter) measures the static pressure inside the DEAA (on the axis) thanks to a lateral opening on the side of the tip (Figure 7A). Considering the volume (estimated from laser line profile sensor Figure 6A) and the pressure of the DEAA, its mechanical cycle as well as its work are estimated (Figure 7B). The oscillations of PDEAA (Figure 7A) result in a complex cycle with several loops (Figure 7B): counter‐clockwise loops provide positive energy (from the DEAA to the flow) while clockwise loops give negative energy; the computed energy is 80 mJ with assistance and 20 mJ without.

As expected, the pressure in the DEAA is lower with voltage compared to the case without (Figure 7A). While the main shape of the cycles in Figure 7B is in accordance with CV–CP cycle of the Figure 5, minor loops are questionable. Without assistance, the DEAA could not provide 20 mJ to the fluid, as it can only dissipate energy (clockwise loops expected). In case of assistance, it is not realistic that the minor loops, induced by the switching from 12 to 0 kV, provides energy to the fluid (counter clockwise loops). Minor dissipating loops (clockwise loops) should be expected. This high frequency component of the cycle comes from disturbance in the static pressure measurement in the DEAA. When the valve is closed and the DEAA contracts, a radial component of the velocity is created which adds a difference of pressure between the center of the tube and the pressure on the DEA's wall (inhomogeneous pressure on the lumen of the DEA). Last but not least, the probe being designed to measure static pressure (on the side of the tip) may be subjected to the effects of this radial velocity (dynamic pressure). Finally, the complex flow induced by these high‐frequency oscillations (high speed and radial flow) prevents us to estimate accurately the pressure on the surface of DEAA (as pointed out by the aforementioned inconsistencies). Thus a direct and accurate quantification of the energy transferred by the DEAA to the fluid is not trivial, especially for the high frequency.

However, it is possible to analyze the energy exchange. This energy transferred from the DEAA to the fluid in its lumen (and surrounding regions) is carried out either through a work of pressure (potential energy) at the outlet/inlet or through the kinetic energy of the fluid going out or in the DEA. Equation based on the energy flux^[^ ^34^ ^]^ (modified Bernoulli's equation) applied in the volume encircled by the DEAA describes the two energy exchanged at the inlet and at the outlet:

(1) $$\begin{array}{c} \frac{dE_{\text{fluid}}}{dt}=\left(P_{\text{in}}v_{\text{in}}-P_{\text{out}}v_{\text{out}}\right)S+\frac{1}{2}\rho S\left(v_{\text{in}}^{3}-v_{\text{out}}^{3}\right)S+\frac{\delta W_{DEA}}{dt} \end{array}$$

with *E*_fluid_ the kinetic energy of the fluid inside the tube, *W*_*DEA*_ the mechanical work of the DEAA, *P* the average pressure in the tube cross‐section, *v* the average speed in the cross‐section, *S* is the cross‐sectional area respectively for the inlet and the outlet of the tube (with the respective subscript in and out), and *ρ* the density of the fluid. For an integration over one cycle of Equation (1), the derivative of the kinetic energy *E*_fluid_ will disappear (no dissipation assumed). Finally, the term resulting from the mechanical work provided by the DEAA (Figure 5) goes to potential energy (right hand first term) and the kinetic energy (right hand second term), both due the fluid conveyed at the inlet and at the outlet of the DEAA. When the pressure is homogeneous along the aorta (quasi‐static transformation), such as in the CV–CP cycle, the energy is only transferred as potential energy (i.e., 50 mJ of area circumscribed by the red line in Figure 5). A difference of pressure due to the rapid expansion or contraction of the tube (CV–CV cycle) will bring kinetic energy as well, since the fluid will be accelerated. In the case of a fast switching, the kinetic energy released goes up to 100 mJ when switching from 12 to 0 kV and up to 10 mJ when switching from 0 to 12 kV (i.e., areas circumscribed by the blue line in Figure 5).

The kinetic energy released by the DEAA in the flow loop triggered dampened pressure oscillation in the lumen of the DEAA and in the compliant chamber (Figure 7A). Oscillations are always related to an exchange of energy between kinetic and potential (elastic) energy. Most of the kinetic energy (100 mJ over 110 mJ in the case of a fast switching) is released when switching from 12 to 0 kV. In that case, the valve is closed and the kinetic energy of the fluid is exchanged with the elastic energy due to the deformation of the DEAA on one side and the compression of the air inside the compliant chamber on the other side (Figure S17A, Supporting Information). It is like an oscillating flow in a U‐tube where the gravity has been replaced on one side by the compression of air in the compliance chamber and by the DEAA on the other side. As soon as the kinetic energy released by the DEAA is completely transferred in the deformation of both the DEAA and of the compliance chamber (i.e., the speed is null), the amplitude of the deformation (related their pressure and volume) can be used to estimate the kinetic energy released by the DEAA (Figure S17, Supporting Information). The estimated 200 mJ, while the quasi static analysis shows a maximum of 100 mJ (Figure 5), shows that this method grasps the oscillating mechanism, but it is not enough to accurately estimate the kinetic energy released by the DEA.

### 3.3 Dielectric‐Elastomer‐Augmented Aorta as a Cardiac Assist Device

**Figure 8:**
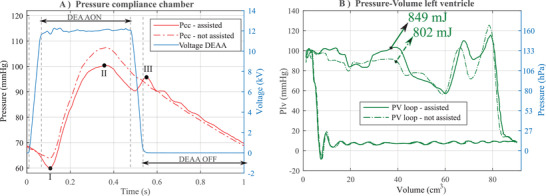
Left ventricle relief thanks to the dielectric elastomer actuator. A) Pressure in the compliance chamber (aorta): The activation of DEAA expands the aortic model just before the start of the systole, resulting in a reduction of the minimum of Pcc (or end‐diastolic pressure, point I) from 65 to 60 mmHg and a lower maximum pressure during systole (or peak systolic left ventricular pressure, point II) from 107 to 100 mmHg. After the closure of the aortic valve, the DEAA (switched‐off) returns to its original diameter, which leads to a diastolic augmentation (point III). B) Pressure–volume cycle of the left ventricle chamber (average of ten cycles). Energy values are provided for assisted and non‐assisted condition

In regard to the heart, the pressures in the left ventricle and in the compliance chamber (the “after‐load” to be overcome by the ventricle) are reduced when DEAA is switched‐on just before the opening of the aortic valve (Figure S20, Supporting Information), compared to the condition without assistance. On the contrary, the pressure in the compliance increases when DEAA is switched off during diastole. To this end, the activation of DEAA expands the aortic model just before the start of the systole, resulting in a reduction of the minimum of Pcc (or end‐diastolic pressure, point I in **Figure** **8**) from 65 to 60 mmHg and a lower maximum pressure during systole (or peak systolic left ventricular pressure, point II in Figure 8) from 107 to 100 mmHg. After the closure of the aortic valve, the DEAA returns to its original diameter, which leads to a diastolic augmentation of 3 mmHg (point III in Figure 8). The flow shows a similar pattern (Figure S16, Supporting Information), characterized by a flow reduction during systole which is compensated by the flow‐increase during the diastole. With or without assistance, the average flow rate in a cycle stays constant (80.6 mL s^−1^ vs 80.4 mL s^−1^ with assistance or without, respectively). The assistance was provided for every heart beat for ten cycles. Figures 8 and 7 considers the periodic state. Results (mean ± standard deviations) are shown in Table S1, Supporting Information, and compared between the two groups (assisted versus not assisted cycles).

The pressure drop seen by the left ventricle when ejecting the flow (afterload) is related to the energy saved. According to the pressure and the volume of the left ventricle on the Figure 8, the work done by the left ventricle is reduced (from 849 to 802 mJ) of 47 mJ thanks to the DEAA, that is, 5.5% of assistance.

The stroke volume of the piston was always kept constant during the experiments.The results showed that the DEAA plugged right after the aortic valve in the flow loop ensures a constant average flow (cardiac output), but at lower working pressures for the heart, that is, lower energy is required to the heart to pump the same amount of volume.

### 3.4 Comparison with Existing Aortic Counter‐Pulsation Devices

The choice of placing “aortic counter‐pulsation devices,” in the ascending aorta, as we did in our in vitro experiment, has been reported^[^ ^35^, ^36^ ^]^ as an option which maximizes cardiac counter‐pulsation effects. Counter‐pulsation in ascending aorta is considered more efficient because: 1) the closer the device is to the aortic valve, the more synchronized the device can be to the cardiac cycle; 2) pulse diffusion is minimized^[^ ^35^ ^]^ (because of the lower pulse spreading in other arteries); 3) blood volume displacement in ascending aorta is enhanced, resulting in more pronounced reduction of the end diastolic pressure

The measurement of the energy of the left ventricle with or without assistance allows a direct analogy with other counter‐pulsation devices. An absolute comparison is difficult due to different working conditions. However, a comparison based on the percentage of assistance shows that we have the same order of magnitude: In humans, Schreuder^[^ ^37^ ^]^ reported a decrease in the left ventricle work (from 4387 mmHg*mL = 583 mJ to 4170 mmHg*mL = 554 mJ) of 29 mJ, that is, 5% of assistance. In calves, Kawaguchi^[^ ^38^ ^]^ reported a decrease (from 914 mmHg*mL = 121 mJ to 849 mmHg*ml = 113 mJ) of 8 mJ, that is, 6.6% of assistance. Our DEAA assist device demonstrates a 47 mJ of assistance (from 848 to 802 mJ, Figure 8B), that is, 5.5% of the energy provided by the human heart (Figure 8).

In the field of cardiovascular medicine, the pressure decrease during the systole induced by any assisting device implanted in humans is seen as a relief of the left ventricle. Even though pressures provide a partial information of assistance, measurements are easier. Kolyva^[^ ^39^ ^]^ reported a diastolic aortic pressure augmentation up to 21.1 mmHg and a reduction of the end‐diastolic pressure from 50.9 mmHg to 43.9 mmHg. In comparison to those values, related to point III and point II of our results in Figure 8, the diastolic aortic pressure augmentation with our DEAA goes up to 3 mmHg while the reduction of the end‐diastolic pressure goes from 65 mmHg to 60 mmHg.

While the working principle is similar, the balloon of the IABP is four to five times longer and it is placed into the descending aorta. IABP with a size of 25 cm length and 16 mm of diameter when inflated, works with a volume change of 40 *cm* ^3^, four times bigger than our device while its size is 5 cm long with only 3.5 cm active and 30 mm of diameter. An increase in the length of the tubular‐DEA could be a simple method to increase the energy provided and/or the pressure drop (or increase). The heart assistance not only being based on the pressure change or on the energy of the left ventricle saved, different strategies of assistance depending on the pathology need to be delved into. Finally, in vivo studies with the extra aortic balloon pump (EABP) and intra aortic balloon pump (IABP) show benefits related to the effect, the position of the device (ascending versus descending)^[^ ^40^ ^]^, the time of activation^[^ ^37^ ^]^ (before or after the closing the valve), and the effect in the coronary.^[^ ^41^ ^]^

## 4 Conclusion

A multilayer dielectric elastomer augmented aorta, for cardiac assist, has been manufactured. Experiment in the flow loop shows that only the potential energy of the DEAA (around 50 mJ) helps the left ventricle. This energy can be transferred thanks to the variation of pressure induced by the heart. Moreover in this proof‐of‐concept study, we demonstrated that a dielectric elastomer actuator is elegant in its cooperation with the heart. On one hand, the risk to rupture blood cells (create haemolysis) and of shear‐induced thrombocyte activation (blood clotting), major problems encountered with other cardiac devices, are removed by augmenting the natural role of the aorta deformation in order to bring energy to the flow. On the other hand, the dielectric elastomer actuator has the benefit of the heart pressure to work efficiently. By direct harness of the controlled stiffness of the DEA, no more elements than the active material are required to make this device light and well integrated.

### 4.1 DEAA Implantation

From the medical point of view, the system presented here shows appealing features. In the event of power failure, our (unactivated) DEAA shows a mechanical response in the same order of magnitude than the natural aorta, providing a viable operating condition for the heart. In the case, the heart has recovered, no further operation will be needed. By avoiding direct contact to the heart, ascending aorta replacement will ease the surgical insertion of the device. The silicone and the carbon‐based silicone electrodes are bio‐compatible. The mechanical connections between the tubular DEA and the aorta can follow the typical surgical procedures (e.g. sewing and gluing) used for existing vascular graft replacements of the ascending aorta^[^ ^42^ ^]^ and for connecting the aorta to active devices such as total artificial hearts^[^ ^43^ ^]^ or para‐aortic counter‐pulsation devices^[^ ^44^ ^]^. However, further developments will focus on optimisation of the junction between the aorta and the device. Last but not least, the DEAA, in future developments, could be triggered either using ECG signals or using the DEAA itself (as both an actuator and a sensor, i.e., self‐sensing)^[^ ^45^ ^]^.

### 4.2 Electrical Energy Transfer

The use of 200 µm thick films eases the fabrication process but requires dedicated electronics with reinforced insulation and a careful electric design. The electrical field, being fully confined in the device, does not represent a risk for tissue damage (despite the 12 kV applied). Due to this, there is a trade off between handling electric insulation and tubular‐DEA fabrication with thinner films. DEA tube based on 100 µm thick film have also been manufactured allowing a working voltage at 6 kV, but the process is longer and not as reliable as with the 200 µm thick film. The use of thinner film requires improvements in the fabrication process. A similar existing process,^[^ ^46^ ^]^ with a film of 18 µm and an electrode thickness of a few hundred nanometers, would reduce, in our case, the voltage down to 1.2 kV, which can be easily manageable. Electrospray deposition^[^ ^47^ ^]^ or organic molecular beam deposition^[^ ^48^ ^]^ can even go below micrometer thick films, but then compliant metal electrodes have to be used in order to support the increase of current^[^ ^49^, ^50^ ^]^. The innovative properties of DEAA technology includes lightweight and the lack of bulky pneumatic drive lines, normally used to actuate the IABP's balloons. Both advantages simplify the possibility of developing fully implanted DEAA device (using trans‐cutaneous wireless power transfer^[^ ^51^, ^52^ ^]^) with the consequent reduction of infections (typical of transcutaneous drivelines). The net electrical energy provided is 224 mJ per cycle (1 s). It corresponds to 224 mW of average power (along the cycle). Considering a 90% efficiency of the power electronic (250 mW) with Li‐ion battery of a cell phone (10 Wh), we can expect an autonomy of 40 h.

### 4.3 DEA Limitations

Apart from material improvement related to its chemistry composition and to the process used to produce the film, the tubular‐DEA has to be designed to have the maximum operating area in the pressure range of the aorta while keeping a safe condition of operation. A fine optimization is difficult for two reasons. First, the breakdown limit is hard to predict as it strongly depends on the deformation^[^ ^53^ ^]^ and on the type of deformation (biaxial or uniaxial). Second, since the DEAA is designed to work close to the plateau (to increase the amount of delivered energy), variability in the fabrication or due to its deformation history is critical. For example, a similar tube we made has reached a mechanical instability at 80 mmHg (12 kV) (Figure S14, Supporting Information) instead of of 105 mmHg (Figure 4). In our approach, we adapted the working pressure range (65–105 mmHg instead of the standard physiological range 80–120 mmHg) to avoid the unstable region. This reduction could be associated to an ill heart but here it is needed to safely operate the designed DEA.

### 4.4 Mode of Actuation

Only the energy related to the CV–CP cycle seems interesting for the left ventricle in our flow loop. Although the mode of actuation suggests the possibility to push the level of assistance up to 160 mJ, our choice of having rising/falling time for DEAA activation/deactivation of 50 ms ensures small and safe oscillation in the flow loop. Faster switching time that releases more kinetic energy is not relevant. Indeed, the two element lump system, that is, the compliant chamber and the total peripheral resistance (TPR), is not reliable to describe the high‐frequency behavior (oscillation) of a real body (continuous media with waves propagation and reflection). In humans, different actuation modes as well as harnessing interaction between the DEAA and complex human circulatory system could be explored to better exploit the total energy that the DEAA could transfer.

## 5 Experimental Section

##### Fabrication of a Module

A module (Figure 3B) was obtained by deposition of an electrode on top of a silicone film (Elastosil film 2030, Wacker, Munich, Germany) with its PET support as provided by the manufacturer. The carbon ink for the electrode^[^ ^54^ ^]^ was deposited on the Elastosil with its support by an automatic film applicator coater and a universal applicator (Zehntner ZAA 2300 and ZUA 2000, Proceq Group, Switzerland) at 20 mm s^−1^ through a mask made with a 13 µm thick PET film (ES301130, Goodfellow) and a 50 µm thick scotch tape (Magic scotch, 3M, SA). Two successive ink depositions are made, giving an ≈10 µm thick electrode after curing for 1 night at 80 °C. Finally, the sheet resistance measured with a two points method is of 4, 8, and 12~*k*Ω.square, respectively, for a uni‐axial strain of 0%, 50%, and 100%.

##### Fabrication of the Multi‐Layer DEA

The manufacturing of the multilayer structure consisted of stacking two modules on top of each other. First, a silicone adhesion layer,^[^ ^16^ ^]^ was deposited with the film applicator coater on a PET substrate (Mylar Typ. A, 125 µm, DuPont Teijin Films, USA) at 20 mm s^−1^ with a profile rod 4.57 um (Zehntner ZSA 2110 Wire‐bar applicator, Proceq Group, Switzerland). The module was reported on the adhesion layer with the profile rod to mimic a laminator. The stack was then put in the oven for 2 h at 80 °C. Finally, the PET supporting the Elastosil film was peeled off. These steps were then repeated to increase the number of layers. On the last Elastosil layer, the last electrode was deposited. At the end, electrodes were interdigited such as in multilayer ceramic capacitor and the stack was removed from its PET support (Figure S9, Supporting Information). The active area was 19 cm long and 35 mm large. The intermediate adhesion layer, ≈5 µm thick did not show difference on the pull‐tester. The number of reported modules was two for 200 µm thick films (or four for 100 µm thick films).

##### Fabrication of the Tube

Being larger than the final size, the stack was cut close to its final size. Before rolling the tube, a silicone layer (Silbione LSR4305, Elkem) was deposited on the bottom of the stack (Figure 2 ⑤; Figure S9, Supporting Information) with the profile rod in order to glue the layers during the rolling. The film, adhering to a poly‐methyl‐methacrylate (PMMA) tube (30 mm diameter) was simply rolled by hand (Figure S10, Supporting Information). The tube was cut across the whole thickness on both edges to reach the different electrodes. The carbon based silicone (Elastosil LR3162 12 gr mixed with 6 mL of Belsil DM 1 Plus, Wacker, Germany) was applied with a syringe at edges of the tube for the electrical connections. A zig‐zag copper wire was bent inside the LR3162 to follow the curvature of the tube. The edges of the wire were abraded using sand paper to improve the adhesion to the carbon‐based silicone and remove the insulating housing of the wire. The tube was then placed in the oven 24 h at 80 °C.

##### Insulation and Shielding

A custom‐made rotating machine was used to perform the outer insulation. When the tube was rotating, silicone (Sylgard 186, Dow) was added with a syringe. Curing was done first at ambient temperature and second in an oven for 2 h at 80 °C. For the inner insulation, the DEA tube was removed from its PMMA support‐tube using iso‐propanol as lubricant. A mold, made of a short PMMA cylinder (30 mm outer diameter, 26 mm inner diameter) and a PET film rolled inside the PMMA cylinder was done (Figure 2 ⑩). The silicone was then injected in between the PET and the PMMA tube and cured for 2 h at 80 °C. The inner and the outer insulation of the high way were at least 2 mm thick (Figure S11, Supporting Information). Finally, carbon ink (same as the one used for the electrodes) was deposited by deep coating on the positive edge to realize the electric shielding, and a coaxial cable (KSIK18HV2619 SPC, Axon, France) was used to connect the positive electrode. The tube was checked using an optical microscope in Figure 3E (Figure S12, Supporting Information, for the 200*μm* thick film).

##### Electrical Measurement

The electrical impedance of the final tubular DEA, measured using an impedance meter (precision impedance Analyzer 4294A, Agilent, USA)was fitted with a capacitance in series with a resistance. For the 100 µm thick film, the capacitance was 6.4 nF and the resistance was 4 *k*Ω, while for 200 µm thick film, the capacitance was 1.6 nF and the resistance 10*k*Ω. Because the tube based on 200 µm thick film was faster to produce and easier to handle, this study was focused on 200 µm thick film based tube, even though realizations based on 100 µm thick film have also been achieved. Regarding the sheet resistance, the value could be improved but a time response of dozens of microseconds was already enough for this application.

##### Quasi‐Static Test Bench

Once produced, the tubular multi layer DEA was then placed in between two coaxial PolyOxyMethylene (POM) tubes (outer diameter 30 mm); Figure 4 shows how DEA's edges are fixed to the POM tubes with elastic tape (polyvinyl tape) all around. On the bottom side of the tube holder, a pressure sensor (PBMN‐258 1 2R A21 44621 2000, Baumer, Germany) was connected while on the top side, the tube was connected to the pneumatic set‐up composed of a dead volume (SMC 1L CP96 I63, Bosch) and a moving piston (SMC CP96 SDL40‐300C, Bosch) driven with a motor and a screw nut system (Rexroth FMS‐080‐SN‐1, Bosch). The dead volume (1 L) gave the capability to stay close to a constant pressure even when the DEAA was on (Figure S15, Supporting Information). The dead volume could also be disconnected to have constant product (pressure × volume = constant, Boyle's law). The pressure was measured via a pressure sensor (PBMN‐258 1 2R A21 44621 2000, Baumer) and using a data acquisition card (NI‐6259, National Instruments, USA). A laser line profile sensor (Gocator 2430A‐2‐R‐01‐S, LMI Technologies) measuring the radial displacement of DEAA was synchronized with the pulses generated by the acquisition card at 100 Hz. By integrating the data of the line profile sensor along the tube and assuming an axisymmetric system, the volume of the tube was estimated. The electrical terminals of the DEAA were connected to a 20 kV voltage amplifier (20/20C‐HS, Trek, USA) through a 20 *M*Ω resistance in order to limit the current in case of breakdown. Finally, the current and the voltage of the DEAA are measured using custom‐made voltage divider and a transimpedance converter with a 10 *k*Ω resistance.

The tube was characterized at low frequency (quasi static), following pressure profile as a triangle wave with maximum pressure reached in 20 s. Before starting the measurement, the tube was mechanically cycled with a pressure significantly above the working pressure as tentative to uniform the tube's history (Mulling effect, Figure S22, Supporting Information). In this work, we did three cycles up to 200 mmHg.

The quasi static test bench was also used to characterize the tube in other operating conditions. For instance, the tube was put under constant pressure while the voltage was switched‐on and ‐off (Movie S2 and Figure S15, Supporting Information).

##### Quasi‐Static Test Bench: Modeling

2D axisymmetric finite elements analysis (COMSOL Multiphysics software, USA) was done using a fully coupled model. Assuming symmetry between the upper part and the bottom part, only half of the tube was simulated. The hyperelastic material model was based on Yeoh's model. To evaluate the parameter of such model, an elastosil silicone membrane was characterized in pure shear configuration (10 cm × 1 cm) on a pull tester at 2 mm s^−1^ (Instron series 3340 with the load cell of 50 N) (Figure S21, Supporting Information). The pure shear deformation was preferred because it was close to the one withstood by the tube. To estimate the parameters of the hyperelastic material model (Yeoh's model with c10 = 0.2 MPa, c20 = 0, c30 = 500 Pa), the measured curve was fitted by introducing the pure shear deformation in the model. Assuming an incompressible material, the bulk modulus was taken high (200 MPa). Membranes and stacked membranes behaved accordingly in the pull‐tester. According to manufacturer (Wacker, Germany) specifications and to the study measurements, a relative permittivity of 2.8 was used in the model. In the simulation, either the pressure or the displacement was imposed in the tube, depending on how close to the instability area.

The simulation of a tubular DEA combines a dual challenge: the electro‐mechanical instabilities and the ones due to the inflation of soft cylinder.^[^ ^31^ ^]^ Those modes were avoided either in simulations or in experiments.

##### Flow Loop

The flow loop (Figure 6A and Figure S18, Supporting Information) was characterized by: an atrium, a left ventricle, a DEA tube, a compliance chamber, and a TPR. A technical drawing is shown in Figure S19, Supporting Information. The valves were mechanical valves clinically used for valve replacement in patients with a nominal diameter of 29 mm (29AGFN‐756, Abbott, IL, USA) and 27 mm (27AGFN‐756, Abbott, IL, USA) were used for mitral (between left atrium and left ventricle chamber, Fig. 7) and aortic valve (between left ventricle chamber and ascending aorta), respectively.

The left ventricle, made of PMMA, was a cubic chamber with side length of 110 mm. The left ventricle chamber was separated by the actuation part by a “silicone membrane” whose expansion/compression was controlled by the piston pump. The volume change of the left ventricle was controlled by the displacement (Figure S20, Supporting Information) of the piston. The controller (model Xemo R294 U, Demcon, Germany) drives the motor (Ecostep‐Motor 34S42, Demcon, Germany) to achieve physiological values at 60 beats min^−1^. The whole setup was filled with deionized water.

The compliance chamber had enclosed a certain air volume which could mimic the arterial compliance, due to air compressibility. Compliance was adjusted by regulating the water level in the chamber. The compliance chamber made of PMMA was 120 mm in length, 110 mm in width, and 290 mm in height. The water level at rest was 181 mm, while the maximum level (with the system running) was around 199 mm. DEA tube was mounted between the left ventricle chamber and the compliance chamber.

The tunable resistance was a custom‐made resistor mimicking the peripheral resistances of the systemic circulation. It consisted of a cylindrical element which had an outlet slot with controllable aperture.^[^ ^33^ ^]^ The flow resistance was measured around 184 Pa.s m^−3^ and the resistance connected the compliance chamber and the atrium chamber with a tygon tube (to close the loop).

##### Flow Loop: Tuning and Measurement in the Flow Loop

Several upgrades were made to the flow loop.^[^ ^32^, ^33^ ^]^ The line profile sensor to measure the radial displacement of DEAA and a pressure‐wire probe (ComboWire XT9515, volcano console, Philips, The Netherlands) 0.36 mm wide were added to characterize the DEA. The static pressure inside the DEA's lumen was measured 1.5 cm from the tip of one side of the wire. The probe measured the pressure on the axis of the DEA. The probe was suspended through a thin PMMA frame (Figure S18, Supporting Information). The probe delay was measured (approximately 8.5 ms). The pressure sensor (PBMN‐258 1 2R A21 44621 2000, Baumer, Germany) in the compliance chamber and in the left ventricle were plugged on the walls of the tanks (Figure 6). In order to measure the flow in the total peripheral resistance, a flow probe (ME16PX, Transonic, USA) was plugged on the tygon tube between the TPR and the left atrium.

The exact operating points of pressure and flow were found by finely tuning the total peripheral resistance, the volume of air in the compliant chamber, and the movement prescribed for the piston (Figure S20, Supporting Information).

The acquisition card (NI‐6259, National Instruments, USA) was initially triggered by the piston displacement, which was driven by a dedicated controller. The acquisition card generated the reference voltage for the voltage amplifier while it measured pressure, voltage, current, and flow. DEAA voltage profile (Figure 7A) had a rising time and falling time of 50 ms, and it was switched on 50 ms before the piston moved forth and switched off 50 ms after it moved back. From the current and the voltage, the energy needed to charge and discharge the DEA was deduced. All tested data were carried out and plotted with Matlab (R2019b).

##### Statistical Analysis

The following signals from flow loop experiments were sampled at 100 kHz for the compliance chamber (PCC), pressure in the lumen of DEA (PDEA), and at 2 kHz for pressure in the left ventricle (PLv), flow in the total peripheral resistance (Q), volume in the left ventricle. The resulting time series were smoothed with a third‐order Savitzky–Golay filter Matlab (Mathworks, Natick, MA, USA) using windows of 99 points. For each cycle, the minimum (PCCmin) and maximum (PCCmax) value of PCC, maximum (Qmax) and average (Qavg) value of Q, and the stroke work (i.e., area of pressure ‐ volume curve in the left ventricle 8) were determined (Table 1). All these values were grouped in two groups: “Assisted group” and not “not assisted group.” The Wilcoxon rank sum test (ranksum) was performed using Matlab (Mathworks, Natick, MA, USA) to compare the values in the two groups. A *p*‐value of less than 0.05 was considered significant.

**Table 1:** Flow loop experiment

	PCCmin [mmHg]	PCCmax [mmHg]	Qmax [mL.s^−1^]	Qavg [mL.s^−1^]	Stroke work [mJ]
Not‐assisted group	65.1 ± 0.5	107.2 ± 0.6	94.2 ± 0.6	80.62 ± 0.3	849 ± 12.7
Assisted group	60.0 ± 0.7	99.8 ± 0.5	91.2 ± 0.5	80.41 ± 0.5	802 ± 16.3
*p*‐Value	0.00004 (*)	0.00004 (*)	0.00004 (*)	0.422	0.00016 (*)

For each cardiac cycle, minimum (PCCmin) and maximum (PCCmax) value of PCC, maximum (Qmax) and average (Qavg) value of Q and the stroke work were determined. Values for each group (not‐assisted and assisted) are expressed as mean ±. For each group *N* = 10 cycles were considered. The Wilcoxon rank sum test (ranksum) was performed to compare the two groups (not‐assisted vs assisted). A *p*‐value of less than 0.05 (indicated by “*”) was considered significant.

## Conflict of Interest

The authors declare no conflict of interest.

## Supporting information

Supporting Information

Supporting Information

Supporting Information

Supporting Information

Supporting Information
