# Supplementary material for: Feasibility of a Dielectric Elastomer Augmented Aorta
Source: Adv Sci (Weinh). 2021 Jan 25;8(6):2001974. doi: 10.1002/advs.202001974 (PMC7967089; doi:10.1002/advs.202001974)
Supplement: Supplementary file 1 — Supporting Information [file ADVS-8-2001974-s002.pdf]

© 2021 Wiley-VCH GmbH

ADVANCED SCIENCE
Open Access

Supporting Information

for *Adv. Sci.,* DOI: 10.1002/advs.202001974

Tubular artificial muscles based on dielectric elastomers for a new generation of cardiac assist devices

Morgan Almanza, Francesco Clavica, Jonathan Chavanne, David Moser, Dominik Obrist, Thierry Carrel, Yoan Civet and Yves Perriard**

Supplementary Materials for

Tubular artificial muscles based on dielectric elastomers for a new generation of cardiac assist devices

Morgan Almanza*, Francesco Clavica, Jonathan Chavanne, David Moser, Dominik Obrist, Thierry Carrel, Yoan Civet and Yves Perriard
*Corresponding author. Email: morgan.almanza@gmail.com

The PDF file includes:

Fig. S9 **Fabrication - Deposition of the adhesion layer**
Fig. S10 **Fabrication - Roll process of the tube**
Fig. S11 **Fabrication - Inner insulation of the tube**
Fig. S12 **Fabrication - Optical sectional view of the tube comprising of 4 layers of 200 μm thick silicone film**

Fig. S13 **Test bench – Measurement of CV-CP cycle**
Fig. S14 **Test bench – Quasi-static measurements of the DEAA without the dead volume**
Fig. S15 **Test bench - Measurement at constant pressure with voltage switching between 0 kV-12kV**

Fig. S16 **Flow loop - Flow rate in the TPR with and without assistance**
Fig. S17 **Flow loop – Study of the oscillation**
Fig. S18 **Flow loop – Photographs of the flow loop**
Fig. S19 **Flow loop - Technical drawings**
Fig. S20 **Flow loop - Voltage applied with the piston displacement and its flow rate.**

Fig. S21 **Mechanical characterization of the elastomer film in pure shear configuration**
Fig. S22 **Mechanical characterization of the elastomer film in pure shear configuration with Mullins effect**
Fig. S23 **Computational model of mechanical deformation of DEA film in pure shear configuration at different electrical field to illustrate the J-shaped and the Banana shape**.

Fig. S24 **Mechanical comparison of a human aorta and the DEAA from uniaxial text**

Fig. S25 Flow loop - Transition in the pressure between unassisted and assisted DEAA

Legend for movies S1, S2, S3, S4

Text S1: **Complementary state of the art of assist device**
Text S2: **Complementary state of the art of existing DEA based pumps**

Other Supplementary Material for this manuscript includes the following:

Movie S1 **Test bench with constant voltage and pressure change applied on the augmented aorta**

Movie S2 **Test bench with constant pressure and voltage switching applied on the augmented aorta**

Movie S3 **Flow loop system**

Movie S4 **Flowloop with the DEAA**

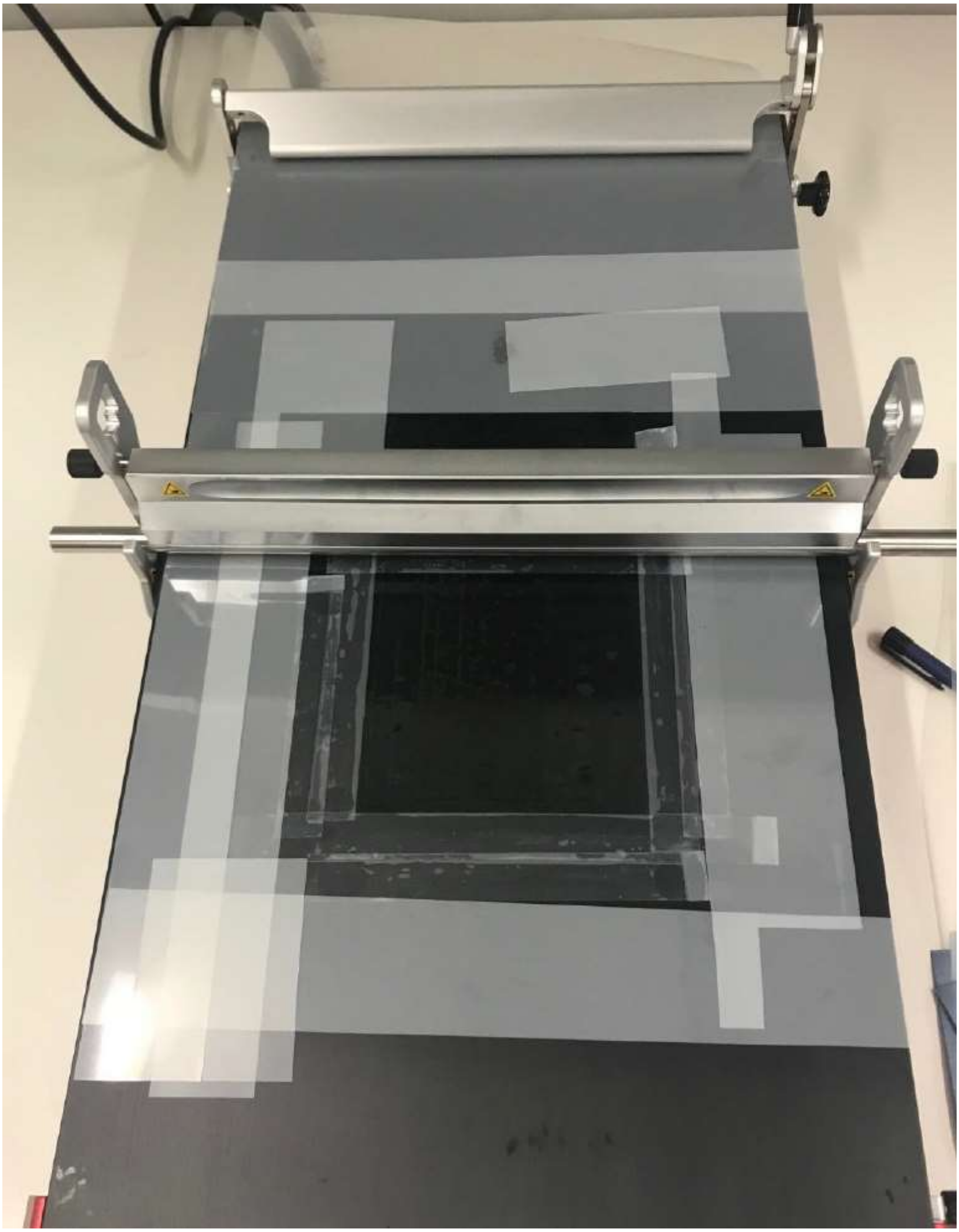

Fig. S9: **Fabrication - Deposition of the adhesion layer.** Photograph of the deposition of the adhesion layer using the automatic film applicator right before the rolling step (Step 5, Fig 2).

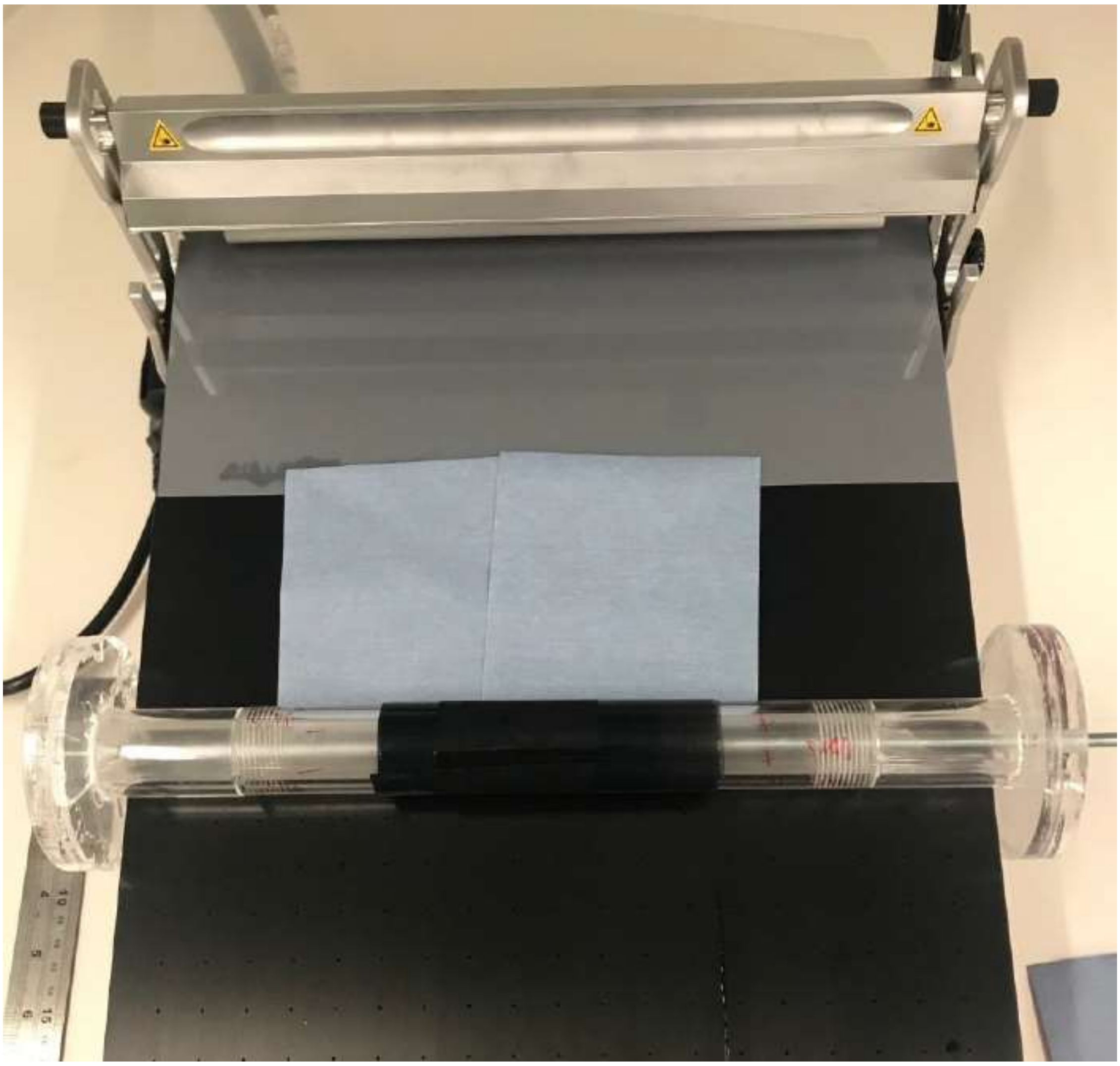

Fig. S10 **Fabrication - Roll process of the DEA**: Photograph of the stack being roll (two turns) around the PMMA tube on top of the automatic film applicator (Step 6, Fig. 2).

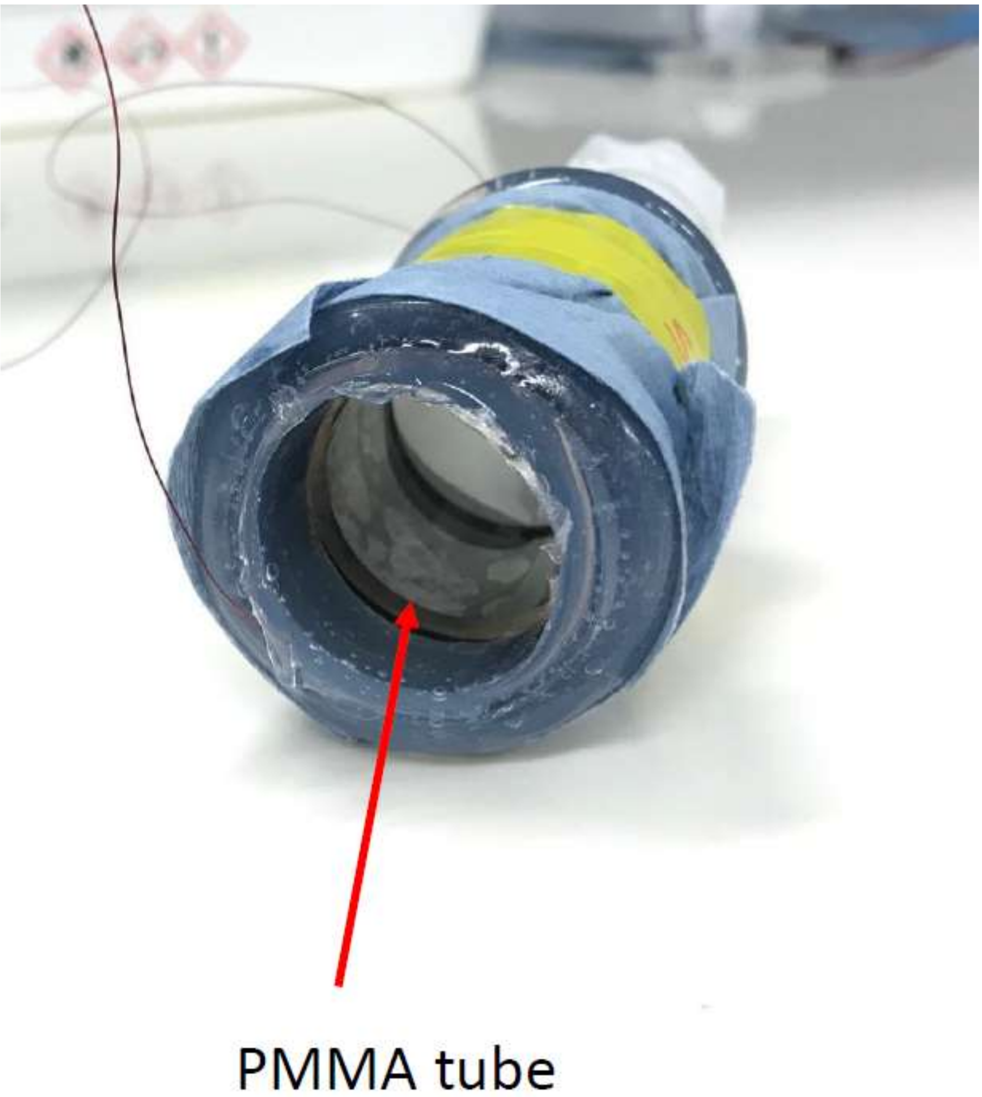

Fig. S11 **Fabrication - Inner insulation of the tube**: Photograph of the tube during the inner insulation step. The inner PMMA tube is visible whereas the PET sheet has been removed for clarity (Step 10, Fig. 2).

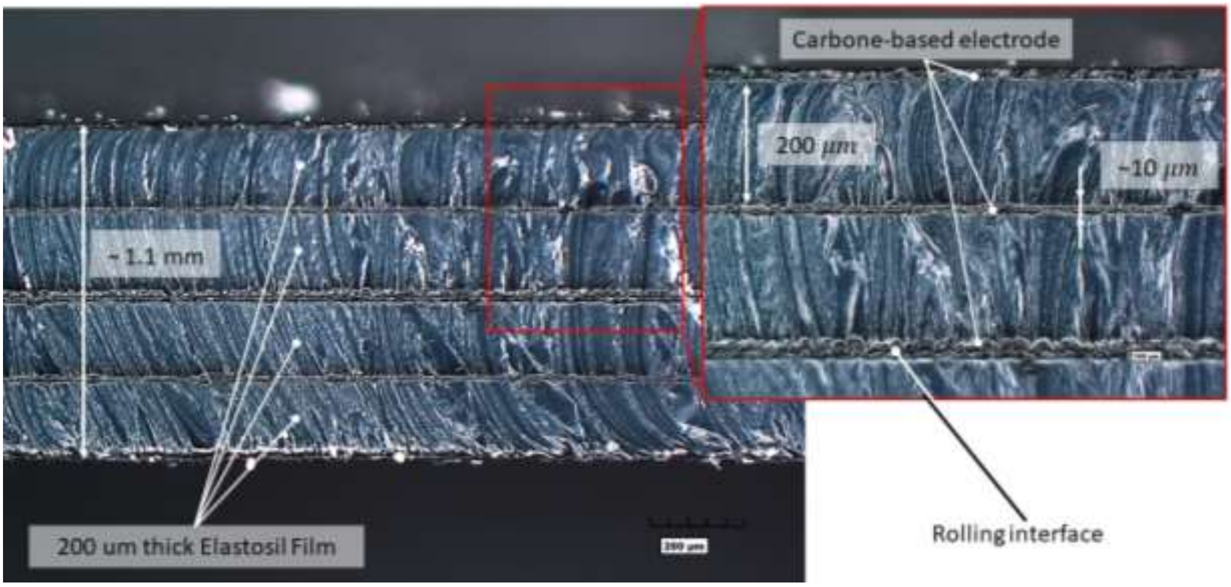

Fig. S12 **Fabrication - Optical sectional view of the tube comprising 4 layers of 200 μm thick silicone film**: Optical cross sectional view of the different layers of a rolled stack. The elastosil film is 200 μm thick.

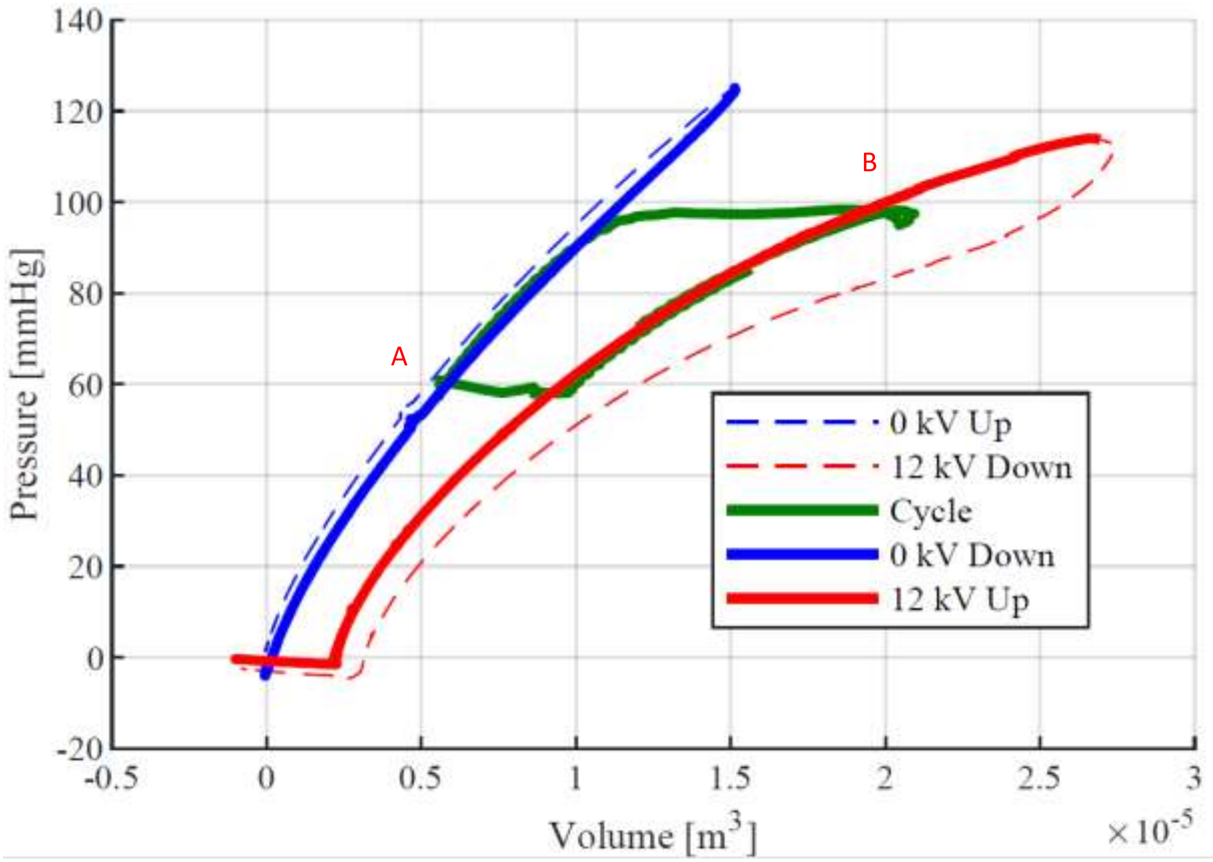

Fig. S13 **Test bench – Measurement of CV-CP cycle on the DEAA** : The red and blue curves show the quasi-static pressure-volume characteristic at 0 kV and 12 kV. The green cycle is obtained in the custom-made test bench by moving the piston back and forth to apply a pressure ranging from circa 60 mmHg to 100 mmHg while switching on the DEAAat minimum pressure (A) and switching off at maximum pressure (B). The characterized tube is not the same as the one used in flow loop or in the Fig. 4.

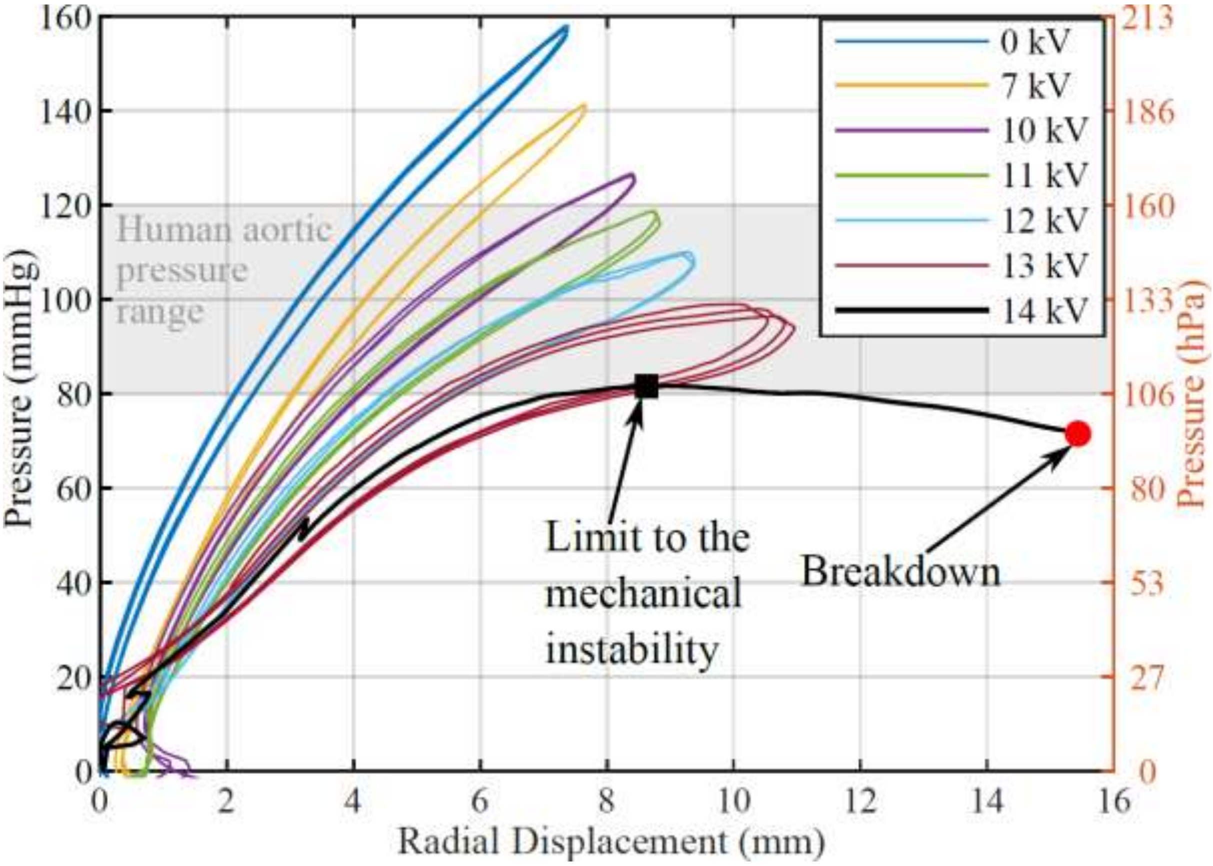

Fig. S14 **Test bench – Quasi-static measurements of the DEAA without the dead volume**. Pressure-displacement characteristic of the DEAA at different voltages according to measurements. The measurements are done in quasi-static conditions with tubular DEA with 4 layers of 200 μm. Removing the dead volume reduced the constant product pressure x volume, thus an increase of volume reduces the pressure.

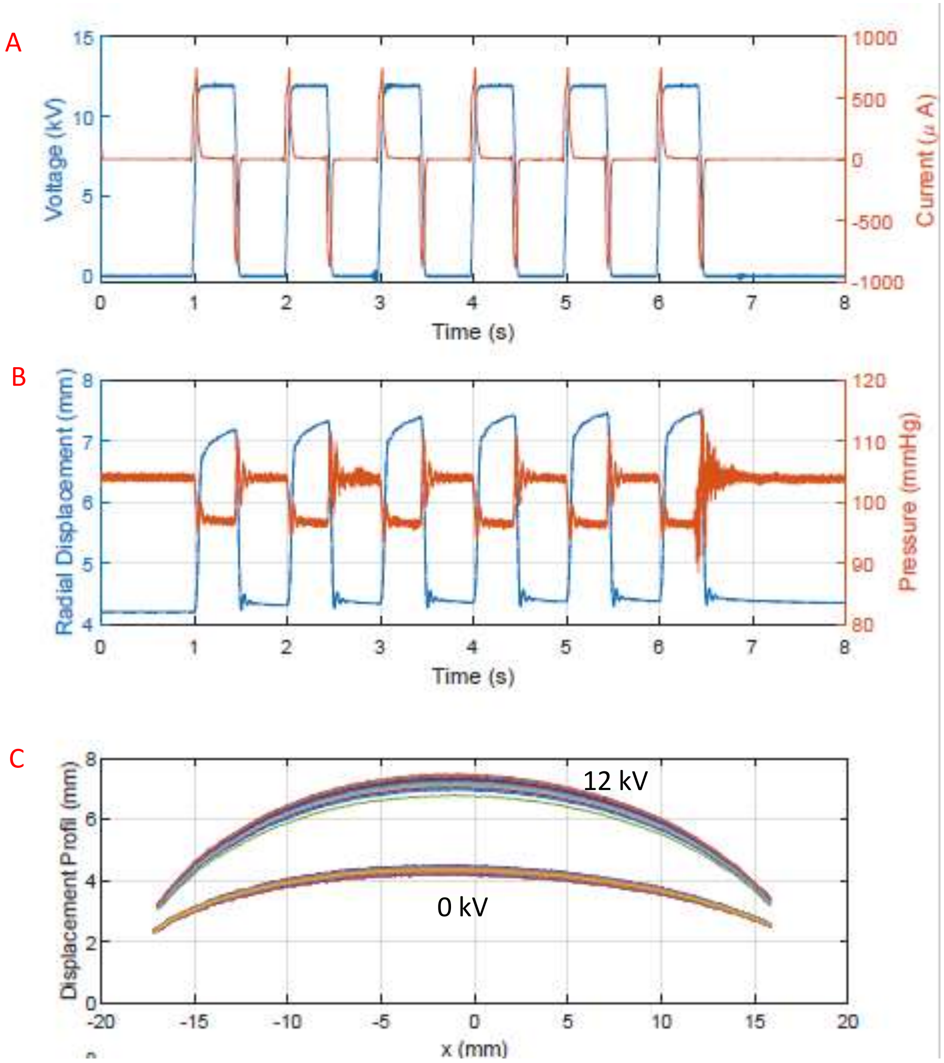

Fig. S15 **Test bench - Measurement at constant pressure with voltage switching 0 kV-12kV of the augmented aorta.** Experimental results of the DEAA considering an initial constant pressure and the activation at a constant voltage. Six voltage steps were conducted in a row. (A) Voltage steps and associated measured current in the DEA. (B) Inner pressure and relative radial displacement measured at the center (apex) of the tube where the displacement is maximal. As soon as the voltage is applied, the DEAA expansion causes a small drop of the pressure according to the volume of the DEAA and of the dead volume (Boyle's law). (C) Displacement profile longitudinally (along the length of the tube) at 0kV and 12kV. The deformation at 0 kV is due to the inner pressure and the displacement at 12kV is a combination of the inner pressure and the Maxwell pressure applied to the membrane of the tube. The displacement have been down sampled from 100 Hz to 10Hz. Adding the 1L dead volume increased the constant product pressure x volume, but the volume being not infinite; we still have some little pressure variation.

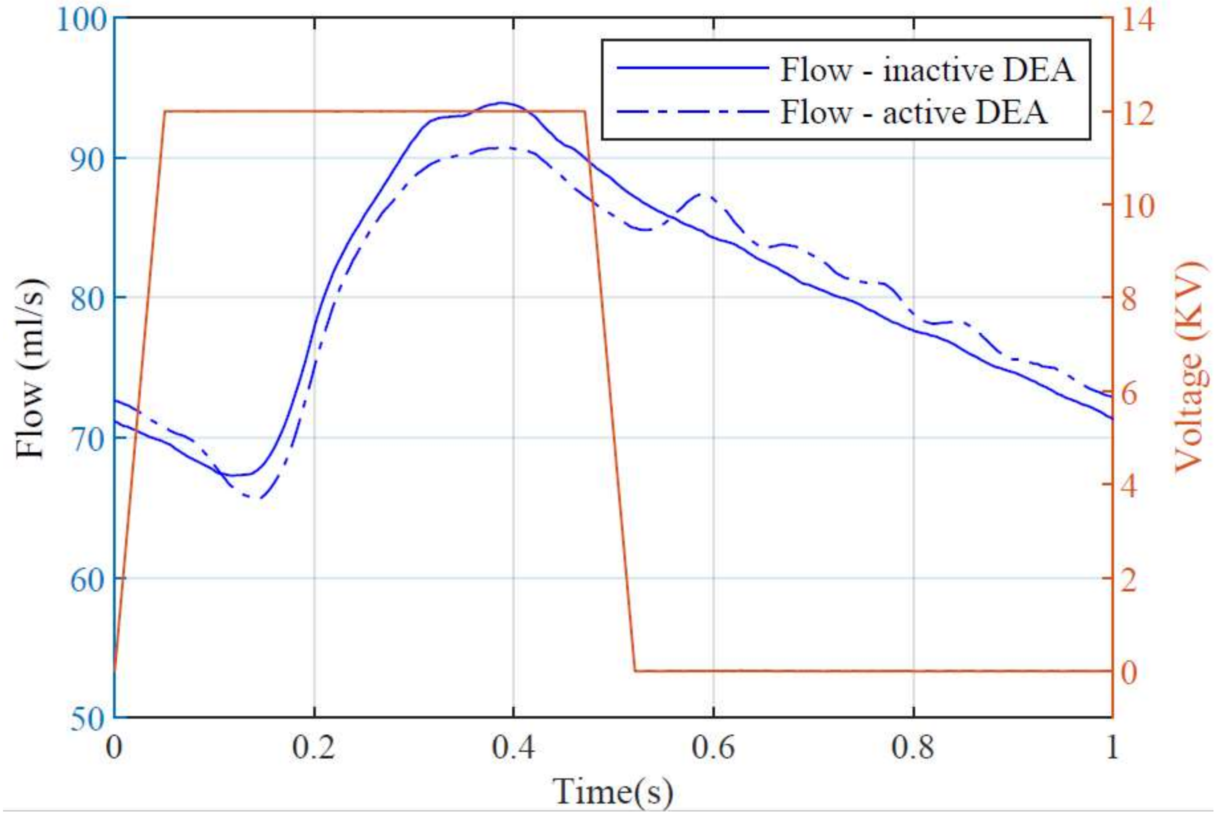

Fig. S16 **Flow loop - Flow rate in the TPR with or without assistance.** Measured flow with DEAA active and inactive. The flow shows a similar pattern characterized by a flow reduction during systole which is compensated by the flow-increase during the diastole. The average flow rate in a cycle stays constant : 80.62ml/sec versus 80.41 ml/sec for DEAA active and not-active, respectively.

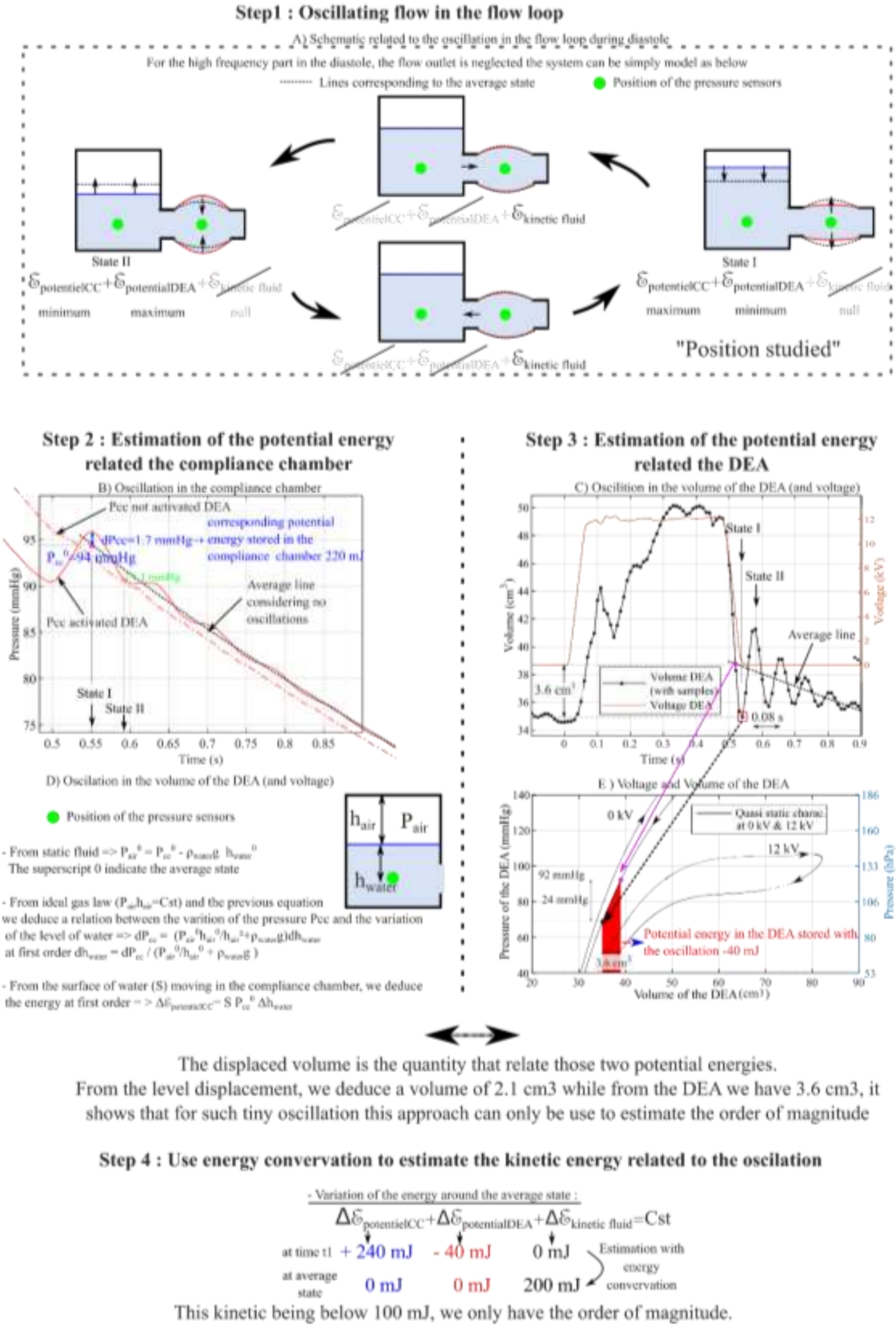

Fig. S17 **Flow loop – Study of the oscillation.** Step 1 Oscillation principle between the DEAA and the compliance chamber, Step 2 estimation of the potential energy stored in the compliance chamber thanks to the oscillation of pressure, Step 3 estimation of the potential energy stored in the DEAA thanks to the oscillation of the volume, Step 4 energy balance to estimate the kinetic energy.

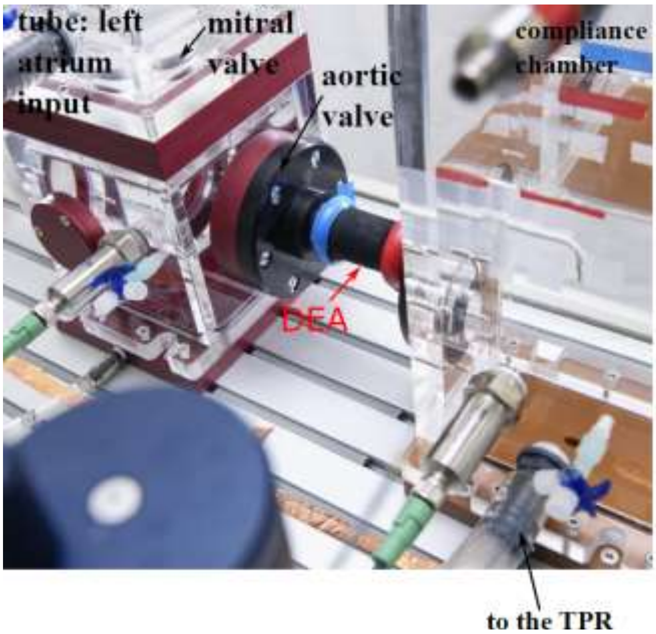

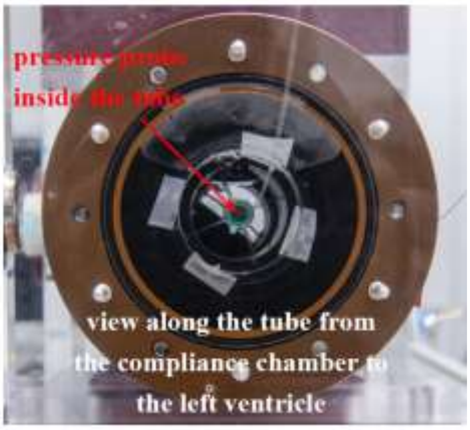

Fig. S18 **Flow loop – Photographs of the flow loop.** (Left Photograph) Back view of the flow loop and (Right Photograph) view along the tube of the DEA

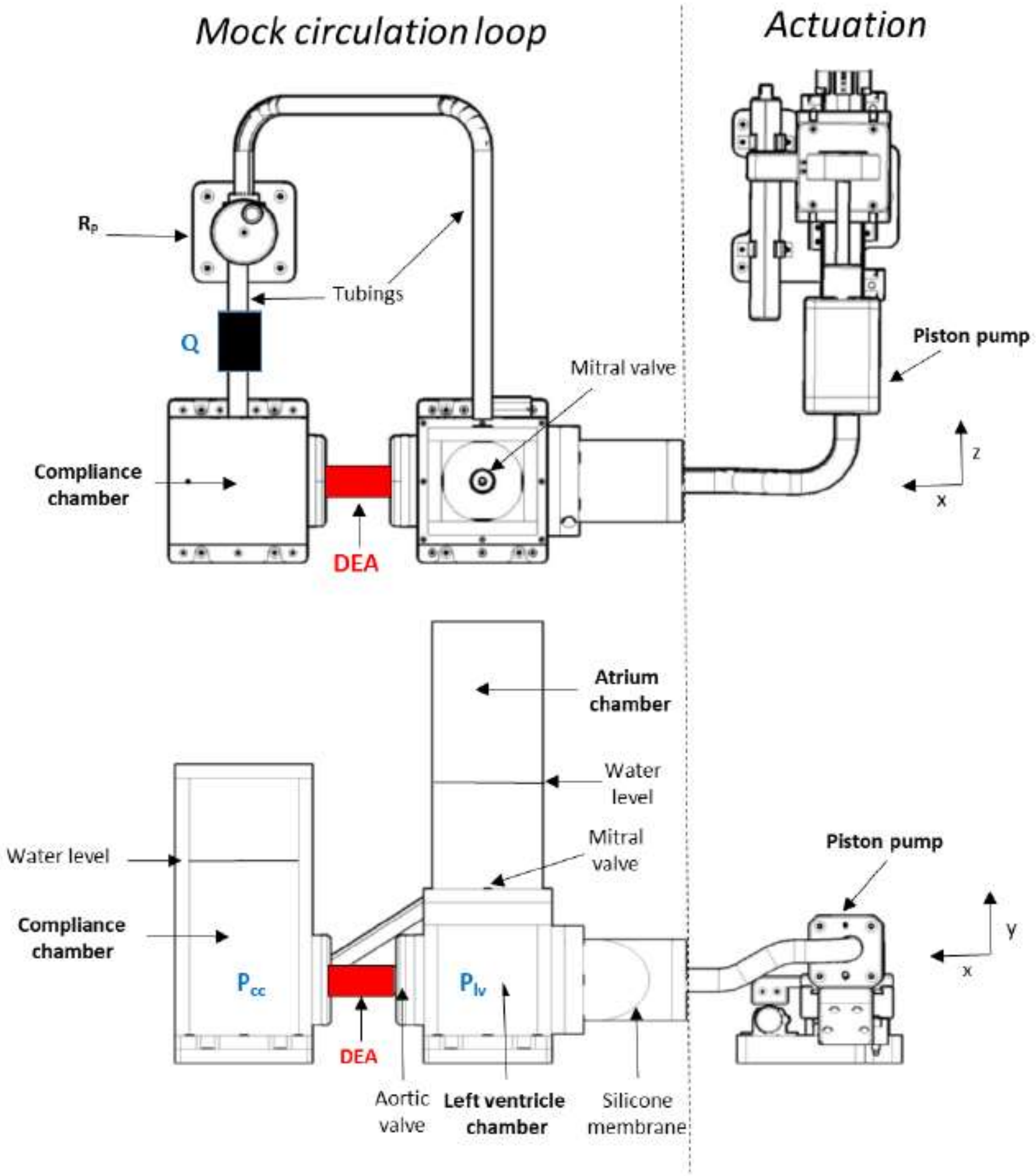

Fig. S19 **Technical drawings of the flow loop.** Technical drawings of the hardware circulatory system: top view of the flow loop (upper panel) and side view (lower panel).

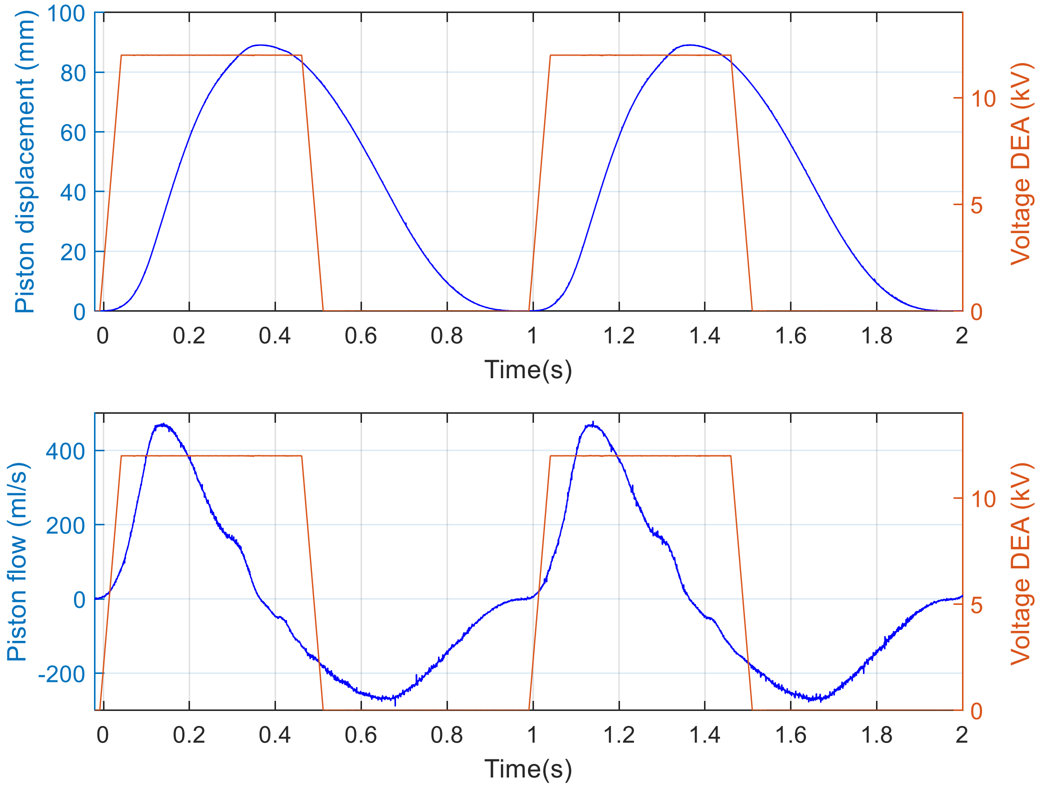

Fig. S20 **Flow loop - Voltage applied with the piston displacement and its flow rate.** The flow is deduced from the position and the diameter of the piston

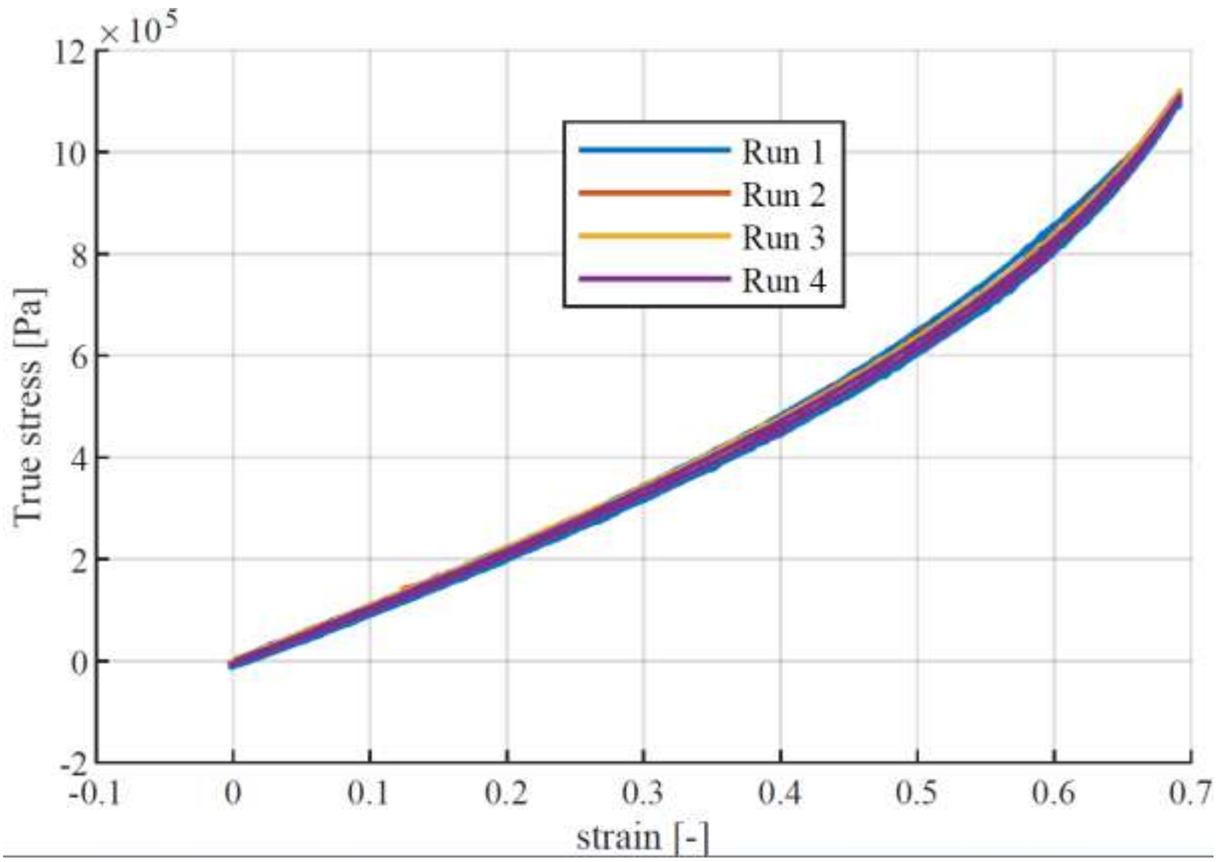

Fig. S21 **Mechanical characterization of the film in pure shear configuration**. Stress-Strain curves of an elastosil sample in pure shear configuration (10cm x 1cm) at 2 mm/s obtained using a pull tester (Instron series 3340 with the load cell of 50 N). Four tests have done on the same sample.

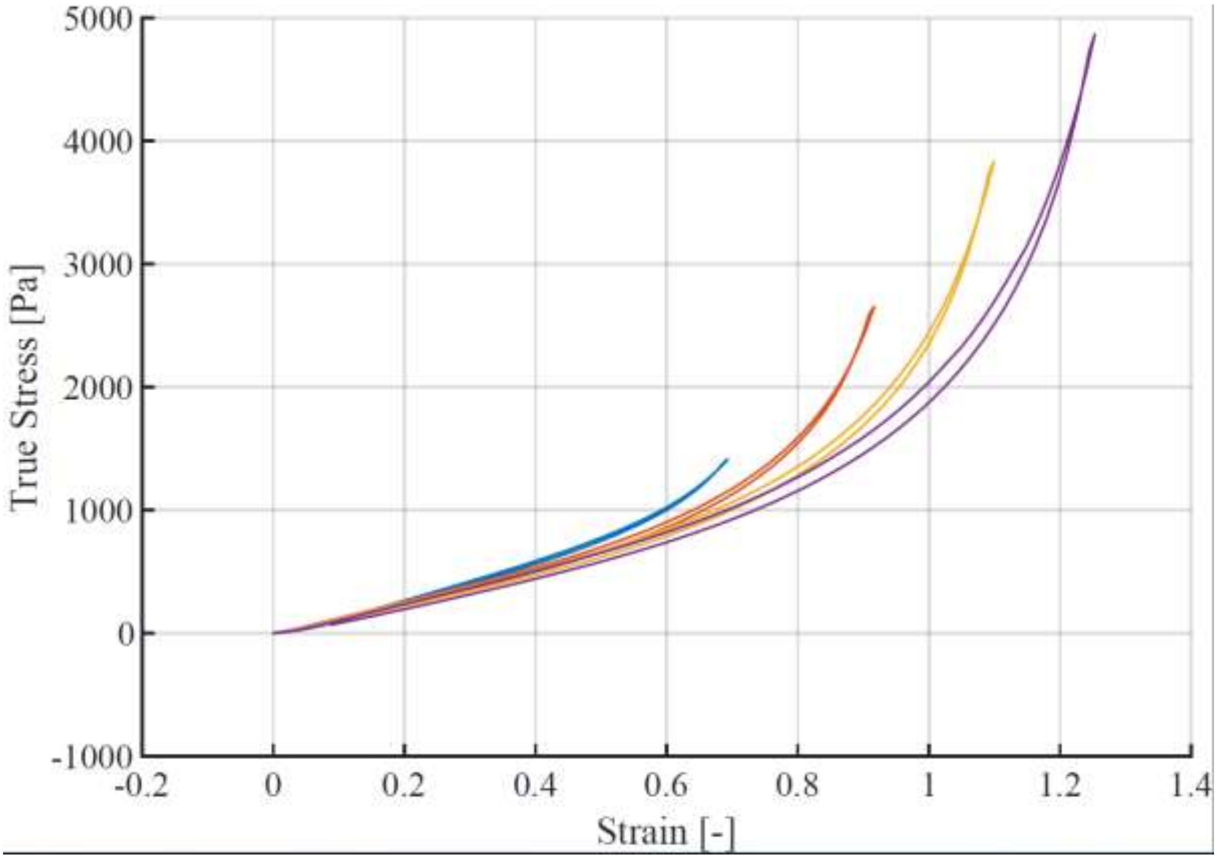

Fig. S22 **Mechanical characterization of the elastomer film in pure shear configuration with Mullins effect**. Stress-Strain curves of an elastosil sample in pure shear configuration (10cm x 1cm) at 2 mm/s obtained using a pull tester (Instron series 3340 with the load cell of 50 N). The same sample has been tested for four different maximum displacements. This highlights the Mullin's effect.

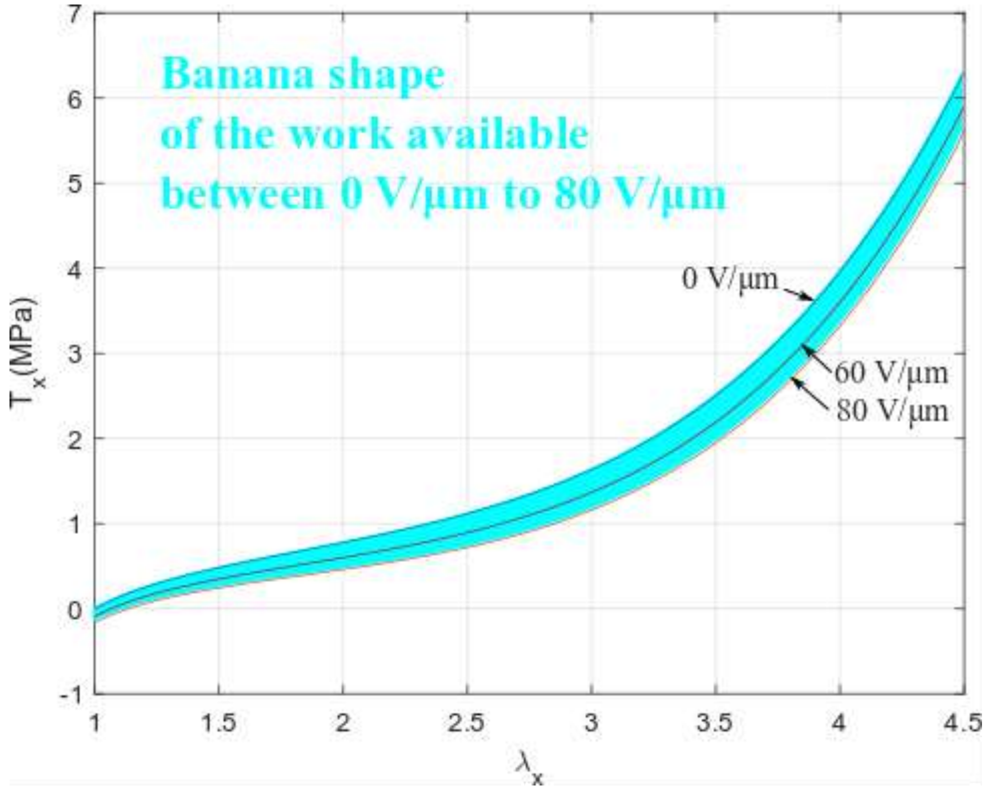

Fig. S23 **Computational model of mechanical deformation (True stress versus stretch) of DEA film in pure shear configuration at different electrical field to illustrate Banana shape**.. The blue area in the banana shape shows the mechanical work available in a conventional DEA. The stress-stretch behavior of the silicone elastomer exhibits a J-shaped curve similar to the natural aorta; however at much greater stretch (4.5 vs 1.3-1.5.)

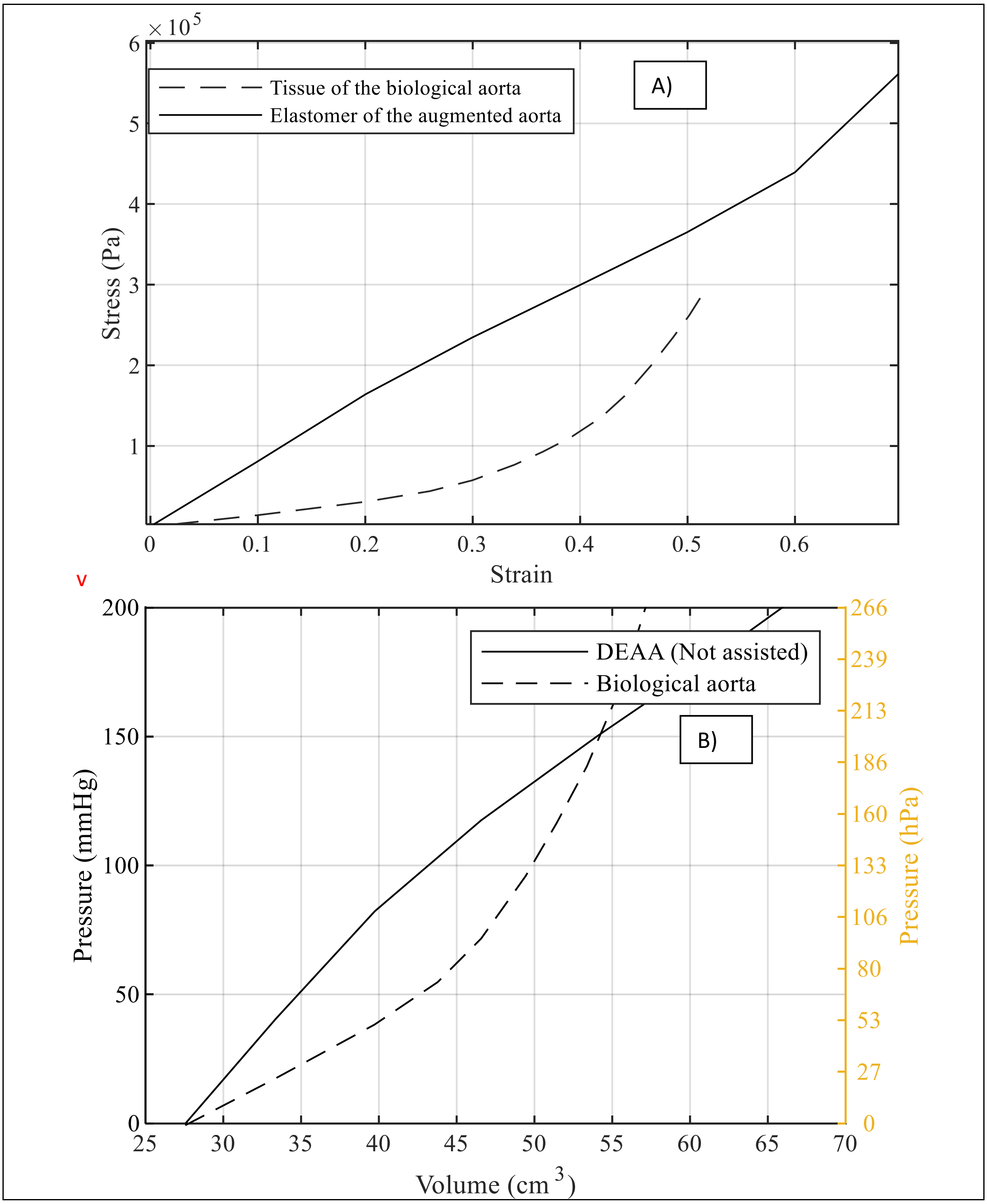

Fig. S24 Mechanical comparison of a natural and the DEAA. A) Comparison in uniaxial configuration (Nominal stress) for silicone film (1 cm x 10 cm at 2 mm/s) (Elastosil 2030) and tissue from a human biological aorta (circumferential direction) based on (Zhalmuratova et al. 2019). B) Comparison in Pressure-Volume diagram extrapolated from the uniaxial test of A) and using Laplace's law. We considered a tube (diameter = 30 mm, length = 39 mm) with a wall thickness of 1mm and 2.5 mm for DEAA and aorta, respectively.

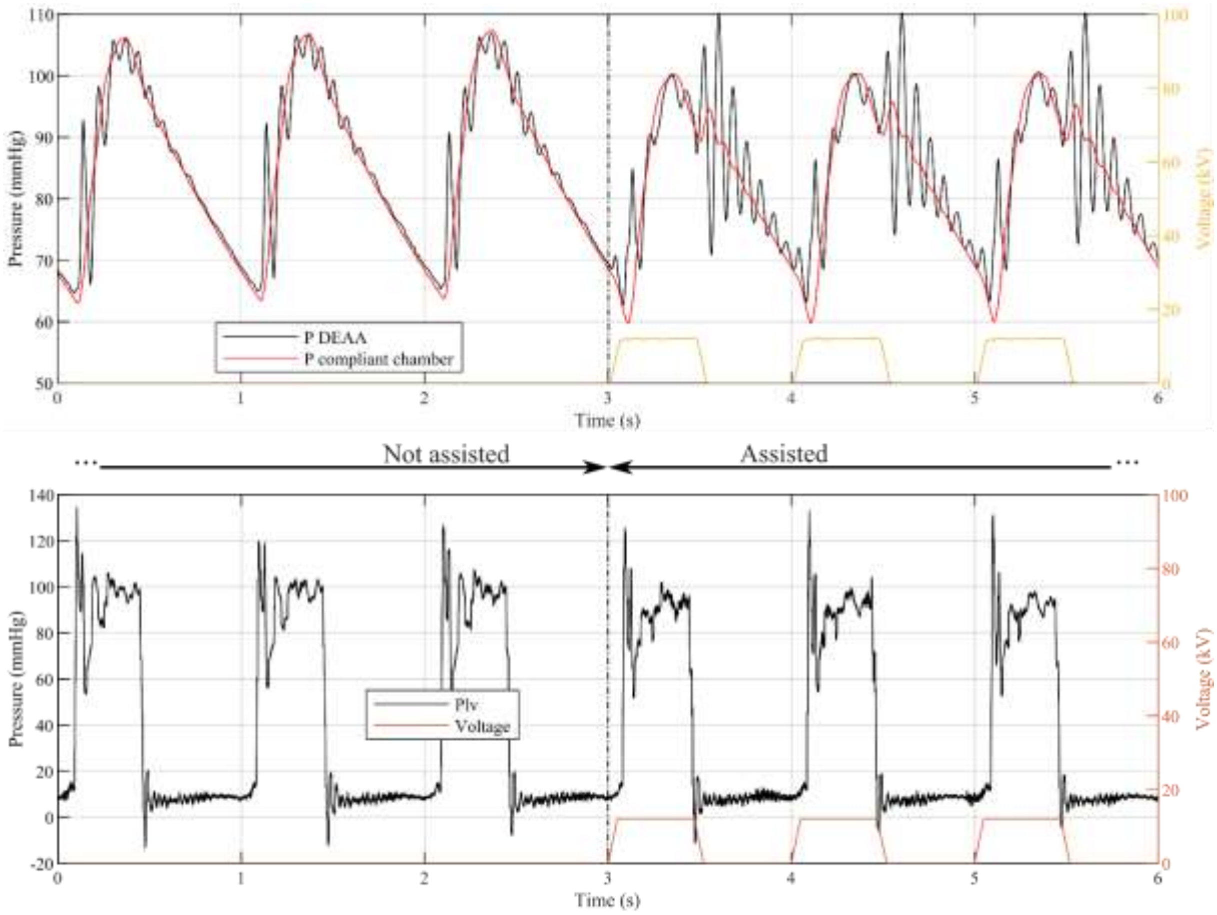

Fig. S25 Flow loop - Transition in the pressure between unassisted and assisted DEAA

<u>Video Legends Video</u>

Movie S1 **Test bench with constant voltage and pressure change applied on the augmented aorta.** The 2D laser measures the profile along the axial length of the tube. The voltage is switched on at the desired voltage. Then, the piston moves slowly forward and then backward. The pressure is measured with the Baumer pressure sensor.

Movie S2 **Test bench with constant pressure and voltage switching applied on the augmented aorta**. Video showing the deformation of the tubular DEA when a constant voltage of 12 kV is applied alternatively. The initial inner pressure is imposed at 105 mmHg. The numerical value of voltage, current, pressure and radial displacement are reported in Fig. S6.

Movie S3 **Flow loop system**. Video showing the pulsatile flow-loop replicating physiological flow and pressure conditions. First, we see the piston pump. Then, we stay a while on the membrane separating the pump and the left ventricle. The red box is the left ventricle with the atrium chamber on top of the latter. We see a silicone aortic root in between the left ventricle and the compliance chamber. The DEAA replaces this aortic root in our proposed working principle. The brown box is the compliance chamber. The tubular vertical black component is the hydraulic resistance. After having an overview of the system, we focus on the aortic valve whose we can see the opening.

Movie S4 **Flowloop with the DEAA**. Test of the DEAA in a “real” condition thanks to the pulsatile flowloop. First, the DEAA is not activated during 4 cycles until 5 s. Then as soon as the DEAAis activated, its deformation increases a lot. The DEAA is activated right before the opening of the aortic valve and deactivated right after the valve closes. We can easily see the high frequency component of the DEAA deformation due to the exchange of potential energy between the compliance chamber and the DEAA during the diastole (i.e. when the pressure decreases, thus when the radial displacement decreases)

Text S1: **Complementary state of the art of assist device**

Technological solutions to assist the heart can be divided into two big families: **ventricular assist devices** (VADs) (including total artificial heart, TAH) and **aortic counterpulsation devices**.

VADs are widely used in clinical setting as destination therapy, bridge to decision, bridge to recovery, bridge to transplantation (Loor and Gonzalez-Stawinski 2012). Current VADs are based on rotary pumps (axial and centrifugal pumps) characterized by a single rotating element which ensures a constant flow (Sen et al. 2016). The rotary pumps have replaced the pulsatile pumps of the 1990s because, compared to the latter, they are significantly simpler (lower number of parts which could fail), smaller in size (less invasive surgery required) and they are characterized by smaller/faster transcutaneous drivelines (electric rather than pneumatic) (Agarwal and High 2012). The main advantages of current VADs are associated to: i) mechanical reliability / possibility of long-term application, ii) fast response (due to electric actuation) and iii) small size (which can be advantageous for the less invasiveness of the surgery and for their potential use in fully implanted pumps). The main limitations of VADS are related to the high risk of haemolysis and thrombosis, due to the high shear generated by the rotating parts (patients must use anti-coagulant throughout their whole lifetime (Heilmann et al. 2009) and the lack of pulsatile flow. Despite the existence of conflicting findings, pulsatile flow is usually preferred to continuous flow as it favours optimal capillary perfusion (Barić 2014)

Aortic-counterpulsation devices can be subdivided in intra-aortic (de Waha et al. 2014, Santa-Cruz, Cohen and Ohman 2006), extra-aortic (Schulz et al. 2016, Davies et al. 2005, Sherwood et al. 2003) and para-aortic (Lu et al. 2011) counterpulsators depending on their location within the aorta. While intra-aortic counterpulsation (i.e. intra aortic balloon pump, IABP) has been widely used in clinical settings for the last 40 years, the applications of extra and para/aortic have been very limited. The basic principle of counterpulsation is similar for all devices: it allows an increase of the coronary blood flow (during diastole) and unloading of the left ventricle (during systole) (de Waha et al. 2014). To this end, a balloon is inflated during the diastolic phase of the heart (which translates into augmentation of the peak diastolic pressure) while presystolic deflation of the balloon decreases the afterload (the pressure against which the heart must pump during systole) (de Waha et al. 2014, Santa-Cruz et al. 2006). IABP is mainly used in high risk patients with severe left ventricular dysfunction as bridge to other solutions (e.g. VADs). The main advantages of IABPs are: i) simplicity to implant (access from femoral artery) ii) pulsatile flow is preserved and ii) anti-coagulation therapy is not required (Pucher et al. 2012). The main complications related to IABP are limb and mesenteric ischemia, bleeding and haemorrhage and infections (Pucher et al. 2012). These complications are associated to the direct arterial access (which also forces the patient to lie in bed) and the size of the pneumatic drivelines (comparing to electric lines). Moreover pneumatic actuation can introduce uncontrollable delays (time for balloon inflaction/deflaction).

Text S2: **Complementary state of the art of existing DEA pump**

Regarding dielectric elastomer based pumping device, circular diaphragm pumps have been already demonstrated (Linnebach et al. 2019; Li, Zhu, et al. 2017). This electrically controlled compliant chamber makes DEA suitable when the inlet pressure allows the deformation of the DE, otherwise a bias elements is required to create suction at zero pressure. On the other hand, DEA peristaltic pump (Li, Wang, et al. 2017; Solano-Arana et al. 2018) and other kinds of DEA impedance pump (Mao et al. 2018), both without additional valves, employ periodic wavelike squeezing and relaxation motions along the tube. Those systems are working independently from the environment, while in the framework of an assist device, the dielectric elastomer actuator has to be integrated in specific working conditions.

Li, Zhe, Yingxi Wang, Choon Chiang Foo, Hareesh Godaba, Jian Zhu, and Choon Hwai Yap. 2017. "The Mechanism for Large-Volume Fluid Pumping via Reversible Snap-through of Dielectric Elastomer." *Journal of Applied Physics* 122 (8): 084503. https://doi.org/10.1063/1.4985827.

Li, Zhe, Jian Zhu, Choon Chiang Foo, and Choon Hwai Yap. 2017. "A Robust Dual-Membrane Dielectric Elastomer Actuator for Large Volume Fluid Pumping via Snap-Through." *Applied Physics Letters* 111 (21): 212901. https://doi.org/10.1063/1.5005982.

Linnebach, P., S. Hau, G. Rizzello, and S. Seelecke. 2019. "Design of a Dielectric Elastomer Actuator Driven Pneumatic Pump." In *Electroactive Polymer Actuators and Devices (EAPAD) XXI*, 10966:109661S. International Society for Optics and Photonics. https://doi.org/10.1117/12.2514034.

Mao, Guoyong, Lei Wu, Yimou Fu, Zhe Chen, Shreyam Natani, Zhe Gou, Xiaodong Ruan, and Shaoxing Qu. 2018. "Design and Characterization of a Soft Dielectric Elastomer Peristaltic Pump Driven by Electromechanical Load." *IEEE/ASME Transactions on Mechatronics* 23 (5): 2132–43. https://doi.org/10.1109/TMECH.2018.2864252.

Solano-Arana, Susana, Florian Klug, Holger Mö\s singer, Florentine Förster-Zügel, and Helmut F. Schlaak. 2018. "A Novel Application of Dielectric Stack Actuators: A Pumping Micromixer." *Smart Materials and Structures* 27 (7): 074008. https://doi.org/10.1088/1361-665X/aac302.

Zhalmuratova, Dinara, Thanh-Giang La, Katherine Ting-Ting Yu, Alexander R. A. Szojka, Stephen H. J. Andrews, Adetola B. Adesida, Chun-il Kim, David S. Nobes, Darren H. Freed, and Hyun-Joong Chung. 2019. "Mimicking 'J-Shaped' and Anisotropic Stress–Strain Behavior of Human and Porcine Aorta by Fabric-Reinforced Elastomer Composites." *ACS Applied Materials & Interfaces* 11 (36): 33323–35. https://doi.org/10.1021/acsami.9b10524.
